# Deletion of TP signaling in macrophages delays liver repair following APAP-induced liver injury by reducing accumulation of reparative macrophage and production of HGF

**DOI:** 10.1186/s41232-024-00356-z

**Published:** 2024-10-03

**Authors:** Mina Tanabe, Kanako Hosono, Atsushi Yamashita, Yoshiya Ito, Masataka Majima, Shuh Narumiya, Chika Kusano, Hideki Amano

**Affiliations:** 1 Department of Pharmacology, Kitasato University School of Medicine, Sagamihara, Japan; 2 Department of Molecular Pharmacology, Graduate School of Medical Sciences, Kitasato University, Sagamihara, Japan; 3 Department of Gastroenterology, Kitasato University School of Medicine, Sagamihara, Japan; 4 Department of Medical Therapeutics, Kanagawa Institute of Technology, Atsugi, Japan; 5 Department of Drug Discovery Medicine, Kyoto University Graduate School of Medicine, Kyoto, Japan

**Keywords:** Acetaminophen, Liver repair, Thromboxane, Macrophage

## Abstract

**Background:**

Acetaminophen (APAP)-induced liver injury is the most common cause of acute liver failure. Macrophages are key players in liver restoration following APAP-induced liver injury. Thromboxane A_2_ (TXA_2_) and its receptor, thromboxane prostanoid (TP) receptor, have been shown to be involved in tissue repair. However, whether TP signaling plays a role in liver repair after APAP hepatotoxicity by affecting macrophage function remains unclear.

**Methods:**

Male TP knockout (*TP*^−/−^) and C57BL/6 wild-type (WT) mice were treated with APAP (300 mg/kg). In addition, macrophage-specific TP-knockout (*TP*^△mac^) and control WT mice were treated with APAP. We explored changes in liver inflammation, liver repair, and macrophage accumulation in mice treated with APAP.

**Results:**

Compared with WT mice, *TP*^−/−^ mice showed aggravated liver injury as indicated by increased levels of alanine transaminase (ALT) and necrotic area as well as delayed liver repair as indicated by decreased expression of proliferating cell nuclear antigen (PCNA). Macrophage deletion exacerbated APAP-induced liver injury and impaired liver repair. Transplantation of *TP*-deficient bone marrow (BM) cells to WT or *TP*^−/−^ mice aggravated APAP hepatotoxicity with suppressed accumulation of macrophages, while transplantation of WT-BM cells to WT or *TP*^−/−^ mice attenuated APAP-induced liver injury with accumulation of macrophages in the injured regions. Macrophage-specific *TP*^−/−^ mice exacerbated liver injury and delayed liver repair, which was associated with increased pro-inflammatory macrophages and decreased reparative macrophages and hepatocyte growth factor (HGF) expression. In vitro, TP signaling facilitated macrophage polarization to a reparative phenotype. Transfer of cultured BM-derived macrophages from control mice to macrophage-specific *TP*^−/−^ mice attenuated APAP-induced liver injury and promoted liver repair. HGF treatment mitigated APAP-induced inflammation and promoted liver repair after APAP-induced liver injury.

**Conclusions:**

Deletion of TP signaling in macrophages delays liver repair following APAP-induced liver injury, which is associated with reduced accumulation of reparative macrophages and the hepatotrophic factor HGF. Specific activation of TP signaling in macrophages may be a potential therapeutic target for liver repair and regeneration after APAP hepatotoxicity.

**Supplementary Information:**

The online version contains supplementary material available at 10.1186/s41232-024-00356-z.

## Introduction

Acetaminophen (APAP; *N*-acetyl-p-aminophenol) is a widely used analgesic and antipyretic drug that is considered safe at therapeutic doses. However, APAP overdose induces severe acute liver injury that progresses to acute liver failure [1]. APAP overdose generates the toxic metabolite *N*-acetyl-p-benzoquinone imine (NAPQI) in hepatocytes in the centrilobular region of the liver. Excessive NAPQI formation causes mitochondrial damage in hepatocytes and subsequently induces mitochondrial oxidative stress [2]. The initial NAPQI-induced direct hepatocyte damage results in the release of damage-associated molecular patterns that trigger a sterile inflammatory response [3]. This response includes the activation of cytokines and the formation of chemokines for the infiltration of immune cells in regions of hepatocyte damage, leading to further aggravation of liver injury in the early phase of APAP toxicity [4, 5]. APAP-induced acute liver injury initiates a regenerative response; however, impairment of the resolution of liver inflammation and liver repair induces persistent liver injury, leading to acute liver failure. Aggravation of acute liver injury and inadequate liver recovery and repair cause high mortality in patients with APAP hepatotoxicity.

Liver macrophages are important for the resolution of liver damage and recovery from APAP-induced liver injury [6]. The infiltrating monocytes in the damaged lesions differentiate into monocyte-derived macrophages (MoMFs). MoMFs are divided into two main subpopulations based on their Ly6C expression levels: Ly6C^high^ and Ly6C^low^. The recruited monocytes expressing Ly6C^high^ differentiate into pro-inflammatory Ly6C^high^ MoMFs (Ly6C^high^ macrophages), and Ly6C^high^ macrophages show pro-inflammatory properties. With the cessation of the inflammatory response, Ly6C^high^ macrophages differentiate into Ly6C^low^ macrophages, which have reparative properties [7]. The macrophage phenotypic shift from a pro-inflammatory to a reparative phenotype at the site of injury is critical [8] because Ly6C^low^ reparative macrophages contribute to the resolution of liver inflammation and restoration of damaged tissues induced by APAP overdose [9]. However, the underlying mechanisms by which liver macrophages contribute to liver repair and regeneration following APAP-induced liver injury remain unknown.

Thromboxane (TX) is an arachidonic acid metabolite and a representative prostanoid. Thromboxane A_2_ (TXA_2_) is produced by the action of cyclooxygenase and TX synthase (TXS) and exerts its activity via the thromboxane prostanoid (TP) receptor [10]. TXA_2_/TP signaling plays an important role in platelet aggregation and smooth muscle contraction. As such, TXA_2_ is involved in vascular diseases of the heart and brain via thrombosis formation and in bronchial asthma via constriction of the bronchial smooth muscle [10]. In the liver, TXA_2_/TP signaling contributes to injury elicited by ischemia/reperfusion and endotoxins [11, 12]. However, recent evidence indicates that TP signaling promotes tissue repair by stimulating angiogenesis in gastric ulcers [13] and ischemic hind limbs [14]. TP signaling is also involved in the resolution of inflammation by enhancing lymphatic drainage through newly formed lymphatic vessels in the mouse tail during secondary lymphedema [15] and in the diaphragm during chronic peritonitis [16]. Furthermore, TP signaling plays a role in liver repair after carbon tetrachloride-induced liver injury [17, 18]. These findings suggest that TXA_2_/TP signaling promotes liver tissue repair following APAP hepatotoxicity by affecting macrophage function.

Therefore, the present study aimed to examine whether TP signaling in macrophages plays a role in liver repair following APAP-induced liver injury in mice.

## Methods

### Animals

Male thromboxane receptor knockout (*TP*^−/−^) mice (8 weeks old) were generated as described previously [19]. Eight-week-old male C57BL/6 wild-type (WT) mice were purchased from CLEA Japan (Tokyo, Japan). *TP*-floxed mice were generated as previously described [16]. Mice with *Tp* deletions in myeloid cells (C57BL/6 background) were generated by crossing *TP*-floxed homozygous mice with LysM-Cre mice. In this study, LysMCre-*Tp*-floxed mice were referred to as *TP*^△mac^ mice, whereas littermate control mice were referred to as control mice. Mice ubiquitously expressing green fluorescent protein (GFP) were kindly provided by Dr. Okabe (Genome Information Research Center, Osaka University, Osaka, Japan). All mice were maintained under controlled humidity (50 ± 5%) and temperature (25 ± 1°C) with a standard light/dark cycle of 12/12 h and were given ad libitum access to food and water.

The experimental protocols were reviewed and approved by the Institutional Animal Care and Use Committee of the Kitasato University School of Medicine (Approval no. 2023–062). All experimental studies were performed in accordance with the institutional guidelines for animal experimentation, based on the Guidelines for Proper Conduct of Animal Experiments published by the Science Council of Japan.

### Animal procedures

The animals were fasted overnight and injected intraperitoneally (i.p.) with 300 mg/kg APAP (Sigma-Aldrich, St. Louis, MO, USA) dissolved in warm pyrogen-free saline (final concentration, 20 mg/mL). At the indicated times, the mice were anesthetized with isoflurane (Pfizer, Manhattan, NY, USA), and blood was drawn from the heart. Liver tissues were collected, a small section of each liver was placed in 10% formaldehyde, and the remainder was immersed in RNAiso Plus reagent (Takara Bio, Shiga, Japan) for further analysis. Following this procedure, the animals were euthanized by cervical dislocation, and death was verified by the lack of heartbeat, respiration, and corneal reflex. Liver injury was determined by measuring serum alanine aminotransferase (ALT) using a Dri-Chem 7000 Chemistry Analyzer System (Fujifilm, Tokyo, Japan).

### Treatments

Ozagrel sodium (Takata Pharmaceutical Co., Saitama, Japan), a TXS inhibitor, or vehicle (phosphate-buffered saline [PBS]) was administered (150 mg/kg, i.p.) [13] 2 h after APAP treatment. ANG-3777 and hepatocyte growth factor (HGF) mimetics (Selleck, Houston, TX, USA) (50 mg/kg) [20] were dissolved in 0.2 mL PBS containing 4% dimethyl sulfoxide and intraperitoneally administered 2 h and 24 h after APAP administration. In a separate experiment, concanavalin A (ConA) (15 mg/kg) dissolved in saline was intravenously (i.v.) injected via the tail vein [21].

#### Clodronate

Clodronate and control liposomes were purchased from FormuMax Scientific (Sunnyvale, CA, USA). Clodronate or control liposomes (100 μL/mouse) were injected intraperitoneally 48 h before APAP administration.

### Histology and immunohistochemistry

Excised liver tissues were fixed immediately with buffered 10% formaldehyde in 0.1 M sodium phosphate buffer (pH 7.4). Sections (3.5 µm thick) were prepared from paraffin-embedded tissues and subjected to either hematoxylin and eosin (H&E) staining or immunostaining. Images of the H&E-stained sections were captured using a microscope (Biozero BZ-700 Series; Keyence, Osaka, Japan). The level of necrosis (as a percentage of the total area) was determined in five fields (100 ×) from each animal by measuring the necrotic area relative to the entire histological section using the ImageJ software version 1.50i (National Institutes of Health, Bethesda, MD, USA). The results are expressed as percentages.

#### Proliferating cell nuclear antigen

With regard to proliferating cell nuclear antigen (PCNA) immunohistochemistry, liver sections were stained with a rabbit monoclonal anti-PCNA antibody (Thermo Fisher Scientific, Waltham, MA, USA) at a dilution of 1:200. Immune complexes were detected using Histofine Simple Stain MAX PO (MULTI) (Nichirei, Tokyo, Japan). Images of stained liver sections were captured using a microscope (Biozero BZ-700 Series). The number of PCNA^+^ hepatocytes was counted in five fields (200 ×) per animal using the ImageJ software. The percentage of PCNA^+^ hepatocytes was then calculated, and the results were expressed as percentages.

### Immunofluorescence staining

Liver tissues from APAP-treated mice were fixed in 4% periodate-lysine-paraformaldehyde overnight at 4°C, transferred to 30% sucrose prepared in 0.1 M phosphate buffer (pH 7.2), and stored at 4°C for 3 days, followed by mounting the liver tissue in Tissue-Tek O.C.T. Compound (Sakura Finetek USA, Inc., Torrance, CA, USA) and stored at – 20°C. Liver tissue Sects. (8-µm-thick) were cut and blocked with 1% bovine serum albumin in 0.5% Triton X-100 in PBS. The sections were incubated with a rabbit anti-mouse TP (1:100; Cayman Chemical, Ann Arbor, MI, USA), a rabbit anti-mouse TXS(1:100; Bioss, Woburn, MA, USA), a rat anti-mouse CD68 antibody (1:100; Bio-Rad Laboratories, Hercules, CA, USA), a rat anti-mouse CD41 (BioRad Laboratories), and a rabbit anti-mouse HGF (1:100; Proteintech Group, Rosemont, Il, US,) at 4°C overnight. The sections were then incubated with the following secondary antibodies at 4°C for 1 h: Alexa Fluor 488-conjugated donkey anti-rabbit IgG, Alexa Fluor 594-conjugated donkey anti-rabbit IgG, or Alexa Fluor 647-conjugated donkey anti-rabbit IgG (Molecular Probes, Eugene, OR, USA). Nuclei were stained with 4′-6-diamidino-2-phenylindole (DAPI). Images were obtained using a fluorescence microscope (Biozero BZ-700 Series; Keyence, Osaka, Japan) or a confocal scanning laser microscope (LSM710; Carl Zeiss, Jena, Germany). After labeling, five optical fields (× 200) per animal were randomly selected, and the number of positive cells was counted.

### Bone marrow transplantation

Bone marrow (BM) cells were obtained by flushing the cavities of freshly dissected femurs and tibiae from donor mice (8 weeks old) using PBS. The flushed BM cells from each donor were dispersed by pipetting and resuspended in PBS at a density of 1 × 10^7^ cells/mL. Mice were lethally irradiated (9.0 Gy) using an MBR-1505R X-ray irradiator (Hitachi Medico Co., Tokyo, Japan) with a filter (copper, 0.5 mm; aluminum, 2 mm) to monitor the cumulative radiation dose. Donor BM mononuclear cells (1 × 10^6^) in 200 μL of PBS were transplanted into the tail veins of irradiated mice. Bone marrow transplantations were performed to generate the chimeric mice as follows: WT mice reconstituted with WT BMMs (WT → WT) or *TP*^−/−^ BMMs (*TP*^−/−^ → WT) and *TP*^−/−^ mice reconstituted with WT BMMs (WT → *TP*^−/−^) or TP^−/−^ BMMs (*TP*^−/−^ → *TP*^−/−^). In a separate experiment, donor BM cells were harvested from GFP^+^-WT mice, and GFP^+^-WT BM cells were transplanted into WT mice. The chimeras were treated with APAP for 8 weeks after BM transplantation.

### Measurement of glutathione and glutathione disulfide

The frozen tissues were homogenized and centrifuged to separate the supernatants. Hepatic glutathione (GSH) and glutathione disulfide (GSSG) levels were measured using a spectrophotometric/microplate reader with a GSSG/GSH Quantification Kit (Dojindo Molecular Technologies, Kumamoto, Japan).

### Measurement of TXB_2_

The concentrations of TXB_2_, a stable metabolite of TXA_2_, in liver tissues were measured using a Thromboxane B_2_ ELISA Kit (Cayman Chemical, Ann Arbor, MI, USA).

### Isolation of intrahepatic leukocytes

Animals were anesthetized by i.p. injection of mixed anesthetic agents containing 4.0 mg/kg midazolam (Sandoz, a Novartis division, Basel, Switzerland), 0.75 mg/kg medetomidine hydrochloride (Nippon Zenyaku Kogyo, Fukushima, Japan), and 5.0 mg/kg butorphanol (Meiji Seika Pharma, Tokyo, Japan). The liver was perfused with Hank’s balanced salt solution through the portal vein. Excised livers were incubated in Roswell Park Memorial Institute (RPMI) medium containing 0.05% collagenase (Type IV; Sigma Chemical Co., St. Louis, MO, USA) for 20 min at 37°C. The liver homogenates were filtered through a 70 μm cell strainer. Non-parenchymal cells were purified by density-gradient centrifugation on 33% Percoll (GE Healthcare Life Sciences, Piscataway, NJ, USA) as previously reported [22].

### Flow cytometric analyses

Isolated non-parenchymal cells were incubated with an anti-mouse CD16/32 antibody (TruStain FcX; BioLegend, San Diego, CA, USA) to block nonspecific binding of the primary mAb. Cells were stained with a combination of the following reagents: PE-conjugated anti-CD45 (30-F11; BioLegend, San Diego, CA, USA), APC/CY7-conjugated anti-Ly6G (1A8; BioLegend), PE/Cy7-conjugated anti-CD11b (M1/70; BioLegend), Brilliant Violet 510-conjugated anti-Ly6C (HK1.4; BioLegend), and anti-F4/80 (BM8; BioLegend). Cells positive for 7-aminoactinomycin D (BioLegend) were excluded from the analysis. Samples were analyzed using a FACSVerse cytometer (BD Biosciences, Franklin Lakes, NJ, USA). Data were analyzed using the Kaluza software v2.1 (Beckman Coulter, Brea, CA, USA). We quantified and presented the number of cells normalized to liver tissue weight (cells/g).

### RT-qPCR analysis

Total RNA was extracted from the liver tissues using RNAiso Plus (Takara Bio). cDNA was constructed with 1 μg of total RNA using the ReverTra Ace qPCR RT Kit (TOYOBO Co., Ltd., Osaka, Japan). Quantitative PCR amplification was performed using the TB Green Premix Ex Taq II (Tli RNase H Plus; Takara Bio, Inc. Shiga, Japan). PCR amplification was performed with the following conditions: 95°C for 10 s, followed by 40 cycles at 95°C for 3 s and 60°C for 20 s. The mRNA expression levels were calculated based on the comparative threshold cycle and normalized to glyceraldehyde-3-phosphate dehydrogenase (GAPDH) expression in each sample. The primer sequences are listed in Supplementary Table S1.

### Cell preparation and culture

To generate BM-derived macrophages, BM cells were isolated from the femurs and tibiae of 8-week-old male mice. BM cells were cultured in 6-well plates (1.0 × 10^6^ cells/well) and maintained in RPMI 1640 medium (Gibco, Thermo Scientific, Waltham, MA, USA) containing 10% fetal calf serum and 20 ng/mL macrophage colony-stimulating factor (BioLegend, San Diego, CA, USA), as previously described [22]. On day 7, BM-derived macrophages were stimulated with lipopolysaccharide (LPS) (10 ng/mL; Sigma-Aldrich, St. Louis, MO, USA) and recombinant murine interferon-gamma (IFN-γ) (20 ng/mL; BioLegend) to polarize toward a pro-inflammatory macrophage phenotype or recombinant murine interleukin (IL)-4 (20 ng/mL; BioLegend) and to polarize toward a reparative phenotype in RPMI 1640 medium for 18 h. Cultured BM-derived macrophages were harvested and homogenized in RNAiso Plus (Takara Bio) and mRNA levels were measured using RT-qPCR.

BM-derived macrophages were incubated with U46619 (1 μM; Cayman Chemical) [16], a TP agonist, with or without SQ22536 (100 μM; Selleck) [23], an adenylate cyclase (AC) inhibitor. After 1 h of incubation, BM-derived macrophages were stimulated with IL-4 (20 ng/mL; BioLegend) for 18 h. The cells were then harvested for quantification of mRNA levels using RT-qPCR.

### Adoptive transfer of BM-derived macrophages

Cultured BM-derived macrophages isolated from controls were harvested on day 7. The cultured BM-derived macrophages (1 × 10^6^ cells in 200 μL PBS) were i.v. injected into control and *TP*^△mac^ mice 4 h after the administration of APAP. At 48 h after APAP treatment, serum and liver tissue samples were collected for analysis.

### Statistical analysis

All results are expressed as the mean ± standard deviation. All statistical analyses were performed using GraphPad Prism, version 8 (GraphPad Software, La Jolla, CA, USA). Data were compared between two groups using unpaired two-tailed Student’s *t* tests and between multiple groups using one-way analysis of variance followed by Tukey’s post hoc tests. Statistical significance was set at *p* < 0.05.

## Results

### Exacerbation of APAP-induced hepatotoxicity in TP^−/−^ mice

**Fig. 1:**
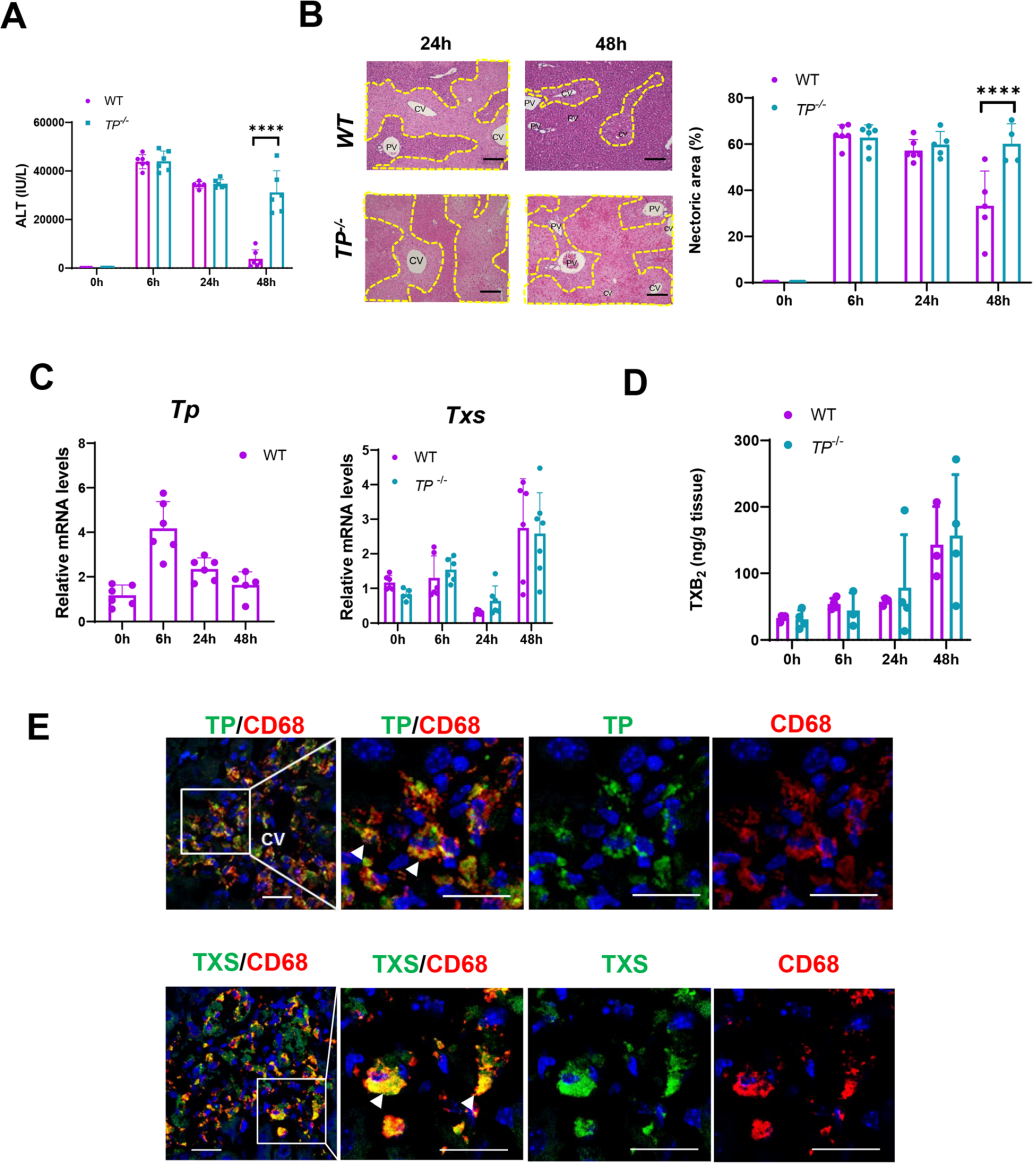
Deletion of TP signaling exacerbates APAP-induced liver injury. **A** ALT levels after APAP treatment in WT and *TP*^−/−^ mice. Data are expressed as the mean ± SD (*n* = 5–6 mice per group). ****p < 0.0001. **B** Representative photos of H&E staining of liver sections from WT and *TP*^−/−^ mice after APAP treatment. The yellow dotted lines indicate areas of necrosis. *CV* central vein, *PV* portal vein. Scale bars: 200 μm. Hepatic necrotic area in WT and *TP*^−/−^ mice after APAP treatment. Data are expressed as the mean ± SD (*n* = 4–6 mice per group). ****p < 0.0001. **C** mRNA levels of *Tp* and *Txs* in the livers from WT and *TP*^−/−^ mice after APAP treatment. Data are expressed as the mean ± SD (*n* = 5–6 mice per group). **D** TXB_2_ levels in the livers from WT and *TP*^−/−^ mice after APAP treatment. Data are expressed as the mean ± SD (*n* = 5–6 mice per group). **E** Double-immunofluorescence staining for TP (green) and CD68 (red) or TXS (green) and CD68 (red). The three right panels are magnified images of the area outlined by the white solid box in the left panel. Scale bar: 20 μm. Arrowheads indicate merged cells. *CV* central vein

To determine the role of TP signaling in APAP hepatotoxicity, we treated both WT and *TP*^−/−^ mice with APAP. Deletion of TP signaling rendered mice highly susceptible to APAP-induced toxicity, as evidenced by increased ALT levels and hepatic necrosis around the central veins (Fig. 1A, B). The levels of ALT peaked at 6 h after APAP treatment in WT mice. Subsequently, ALT levels were reduced after 48 h. By contrast, the levels of ALT and hepatic necrosis remained high in *TP*^−/−^ mice. These results indicate that liver injury was enhanced and liver recovery was delayed in *TP*^−/−^ mice. PCR analysis showed that hepatic levels of *Tp* increased 6 h after APAP treatment (Fig. 1C). Hepatic levels of *Txs* moderately increased in both genotypes 48 h after APAP treatment; however, there was no statistical difference in *Txs* mRNA levels between the two genotypes. Furthermore, the concentration of TXB_2_, a stable metabolite of TXA_2_, was increased in the livers after APAP administration in WT and *TP*^−/−^ mice, and no significant difference between the two genotypes was observed (Fig. 1D). Immunohistochemical staining for TP and TXS demonstrated that TP and TXS were expressed in CD68^+^ cells, suggesting that TXA_2_ synthesized by macrophages bound to TP (Fig. 1E).

Because TP signaling is involved in platelet aggregation, platelet accumulation during APAP hepatotoxicity was evaluated by immunostaining platelets with an anti-CD41 antibody. Immunostaining demonstrated that CD41^+^ cells were extensively observed in the livers treated with APAP for 48 h; however, there was no statistical difference in the CD41^+^ area between the two genotypes (Supplementary Fig. 1).

### Involvement of macrophages in liver repair after APAP-induced liver injury

**Fig. 2:**
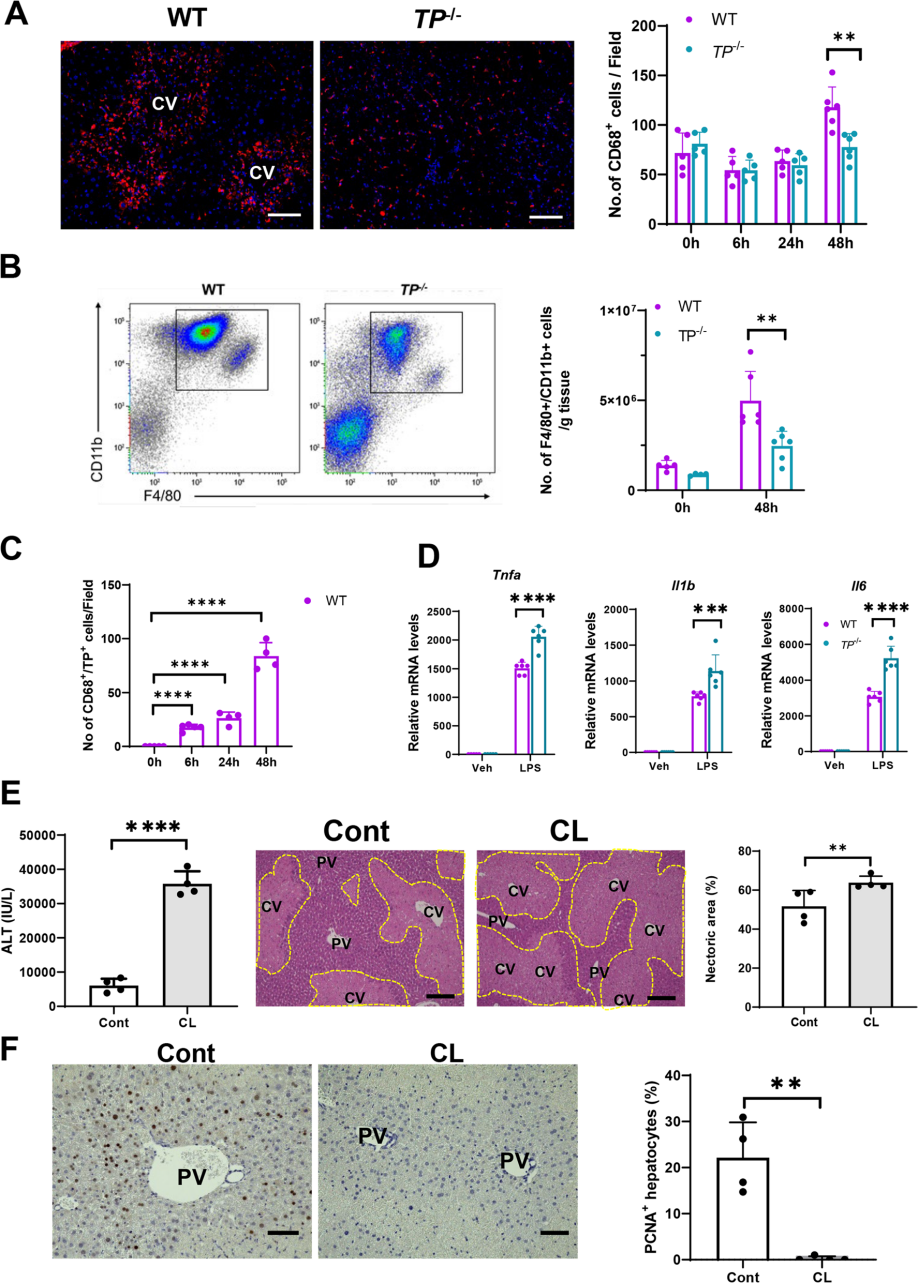
Deletion of macrophages with clodronate liposomes (CL) aggravates APAP-induced liver injury and delays liver repair. **A** Representative microphotographs of immunostaining of CD68 (red) in the livers from WT and *TP*^−/−^ mice 48 h after APAP treatment. *CV* central vein. Scale bars: 100 μm. The number of CD68^+^ cells (macrophages) in the livers from WT and *TP*^−/−^ mice after APAP treatment. Data are expressed as the mean ± SD (*n* = 5–6 mice per group). **p < 0.01. **B** Representative dot plots of liver macrophages (F4/80^+^/CD11b^+^ cells) gated out CD45^+^/Ly6G^high^/CD11b^high^ cells and number of macrophages 0 and 48 h after APAP treatment in WT and *TP*^−/−^ mice. Data are expressed as the mean ± SD (*n* = 5–6 mice per group). **p < 0.01. **C** The number of CD68^+^ macrophages expressing TP (TP^+^/CD68^+^ cells) in the livers from WT mice after APAP treatment. Data are expressed as the mean ± SD (*n* = 4–5 mice per group). ****p < 0.0001. **D** Expression of mRNA encoding genes related to a pro-inflammatory macrophage phenotype (*Tnfa*, *Il1b*,and* Il6*) in cultured BM-derived macrophages from WT and *TP*^−/−^ mice stimulated with LPS for 3 h. Data are expressed as the mean ± SD (*n* = 6 mice per group). ***p < 0.001; ****p < 0.0001. **E** Levels of ALT and hepatic necrotic area 48 h after APAP treatment in WT mice treated with clodronate liposomes (CL) or control liposomes (Cont). Data are expressed as the mean ± SD (*n* = 4 mice per group). **p < 0.01; ****p < 0.0001. Representative images of H&E staining of WT mice treated with CL or Cont. *CV* central vein, *PV* portal vein. Scale bars: 200 μm. **F** PCNA^+^ hepatocytes (%) 48 h after APAP treatment in WT mice treated with CL or Cont. Representative images showing immunohistochemical staining for PCNA in WT mice treated with CL or Cont. *PV* portal vein. Scale bars: 100 μm. Data are expressed as the mean ± SD (*n* = 4 mice per group). **p < 0.01

Because macrophages are responsible for liver repair after APAP-induced liver injury, we assessed the accumulation of macrophages during APAP hepatotoxicity. The number of CD68^+^ cells (macrophages) in WT livers increased 48 h after APAP treatment, and the accumulation of CD68^+^ cells in WT livers was greater than that in *TP*-deficient livers (Fig. 2A). These results suggest that macrophage recruitment was accompanied by liver repair after APAP-induced liver injury. Flow cytometric analysis also demonstrated that the number of F4/80^+^/CD11b^+^ macrophages in WT livers was higher than that in *TP*-deficient livers (Fig. 2B). We further determined changes in the number of CD68^+^ macrophages expressing TP in WT livers using immunofluorescence analysis. The number of TP-expressing macrophages was increased during APAP-induced liver injury (Fig. 2C, Supplementary Fig. 2). To explore the functional differences in macrophages between WT and *TP*^−/−^ mice, we stimulated cultured BM-derived macrophages with LPS for 3 h and determined cytokine mRNA levels. The results showed that mRNA levels of pro-inflammatory cytokines, including *Tnfa*, *Il1b*, and *Il6*, were higher in TP-deficient macrophages than in WT macrophages (Fig. 2D), indicating that *TP*-deficient macrophages have a higher capacity to produce pro-inflammatory cytokines.

To elucidate the role of macrophage accumulation in the restoration of injury to APAP hepatotoxicity, WT mice were pretreated with clodronate liposomes (CL) to delete macrophages or with control liposomes (Cont). The results showed that the levels of ALT and hepatic necrotic area in CL-treated WT mice 48 h after APAP treatment were higher than those in Cont-treated WT mice (Fig. 2E), which was consistent with previous reports [24]. At 48 h after APAP treatment, CL treatment decreased the number of hepatic macrophages (F4/80^+^/CD11b^+^cells) compared to Cont (Supplementary Fig. 3). The expression of PCNA, a marker of hepatocyte proliferation, was considerably lower in the CL-treated mice than in the Cont-treated mice. These results suggested that hepatic macrophages are involved in the resolution of liver inflammation and repair after APAP treatment.

### Deletion of TP signaling in BM-derived macrophages delayed liver repair

**Fig. 3:**
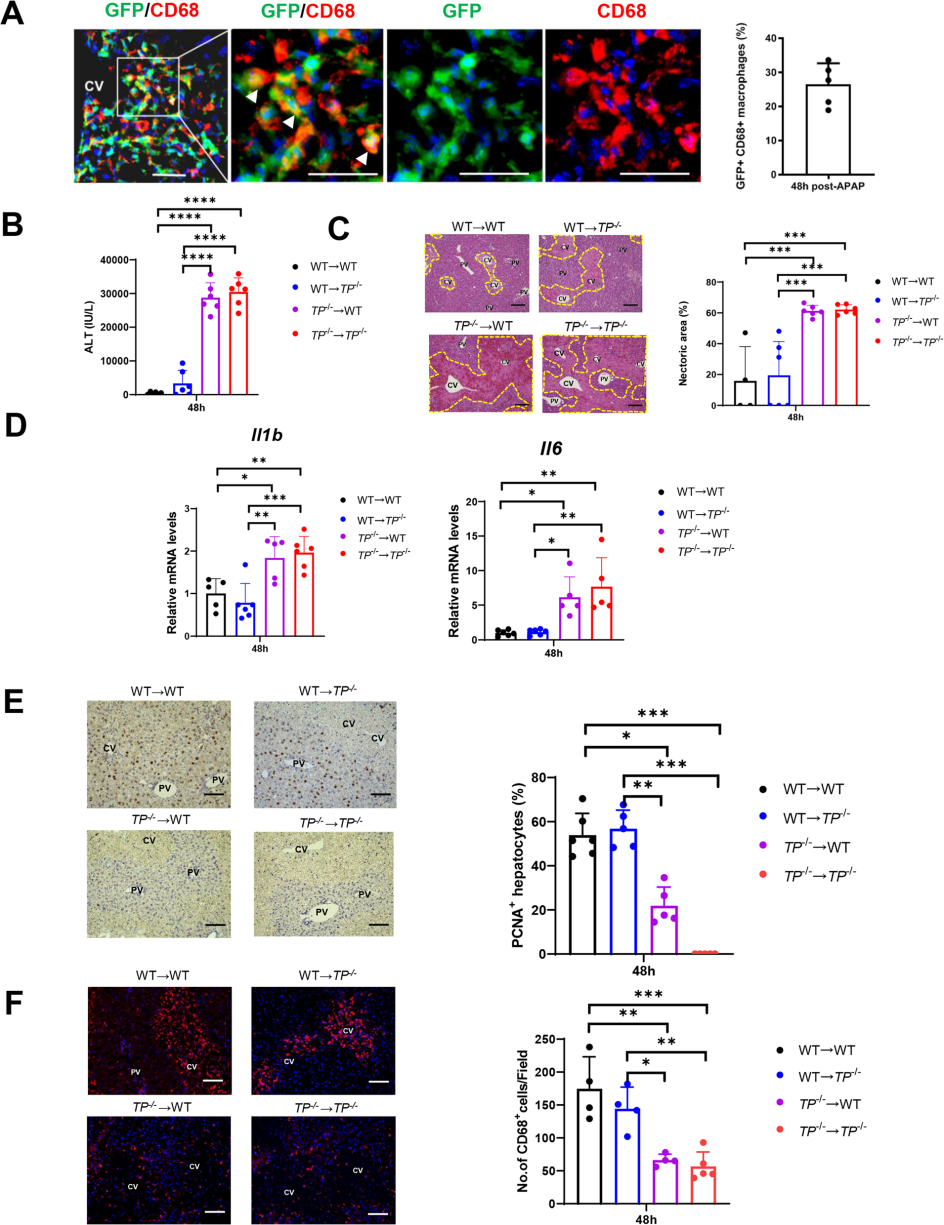
TP signaling deficiency in bone marrow cells exacerbates APAP-induced liver injury and delays liver repair. **A** Representative immunofluorescence images of GFP (green) and CD68 (red) in the liver of WT mice bearing GFP^+^ bone marrow (BM) cells 48 h after APAP treatment. The nuclei were stained with DAPI (blue). *CV* central vein. The three right panels are magnified images of the area outlined by the white solid box in the left panel. Scale bar: 20 μm. Arrowheads indicate merged cells. *CV* central vein. The percentage of CD68^+^ cells positive for GFP in the livers of WT mice bearing GFP^+^ BM cells 48 h after APAP treatment. Data are expressed as the mean ± SD (*n* = 5 mice per group). **B** ALT levels 48 h after APAP treatment in BM chimeric mice. Data are expressed as the mean ± SD (*n* = 6 mice per group). **C** Representative images showing H&E staining of liver sections. *CV* central vein, *PV* portal vein. Scale bars: 200 μm. Hepatic necrotic areas 48 h after APAP treatment in BM chimeric mice. Data are expressed as the mean ± SD (*n* = 4–6 mice per group). ***p < 0.001. **D** Expression of mRNA encoding *Il1b* and *Il6* in the liver 48 h after APAP treatment in BM chimeric mice. Data are expressed as the mean ± SD (*n* = 5–6 mice per group). *p < 0.05; **p < 0.01; ***p < 0.001. **E** Representative images showing immunohistochemical staining of PCNA at 48 h after APAP treatment in BM chimeric mice. *CV* central vein, *PV* portal vein. Scale bars: 100 μm. Percentage of PCNA^+^hepatocytes in BM chimeric mice. Data are expressed as the mean ± SD (*n* = 5–6 mice per group). *p < 0.05; **p < 0.01; ***p < 0.001. **F** Representative images showing immunofluorescence staining for CD68 48 h after APAP treatment in BM chimeric mice. *CV* central vein, *PV* portal vein. Scale bars: 100 μm. Number of CD68^+^ macrophages in the BM of chimeric mice. Data are expressed as the mean ± SD (*n* = 4–5 mice per group). *p < 0.05; **p < 0.01; ***p < 0.001

To examine whether the macrophages accumulated in the injured regions were derived from the BM, we generated chimeric WT mice bearing GFP^+^ BM cells. Immunofluorescence demonstrated that accumulated GFP^+^ cells in the injured regions colocalized with CD68^+^ cells (approximately 30% of CD68^+^ cells were positive for GFP) 48 h after APAP treatment (Fig. 3A), indicating that the infiltrating macrophages were derived from the BM. To further define the role of TP-expressing BM cells in liver repair after APAP treatment, we performed BM transplantation and assessed ALT levels and hepatic necrosis in response to APAP treatment. Reconstitution of WT mice with *TP*-deficient BM (*TP*^−/−^ → WT mice) resulted in enhancement of APAP-induced liver injury, as indicated by increased levels of ALT (Fig. 3B) and hepatic necrosis area (Fig. 3C). The magnitude of liver injury in WT mice transplanted with *TP*-deficient BM was equivalent to that in *TP*^−/−^ mice transplanted with *TP*-deficient BM (*TP*^−/−^ → *TP*^−/−^ mice). By contrast, the levels of ALT and hepatic necrotic area in WT → WT and WT → *TP*^−/−^ mice were lower than those in *TP*^−/−^ → WT and *TP*^−/−^ → *TP*^−/−^ mice. The mRNA levels of pro-inflammatory cytokines including *Il1b* and *Il6* in WT → WT and WT → *TP*^−/−^ mice were lower than those in *TP*^−/−^ → WT and *TP*^−/−^ → *TP*^−/−^ mice (Fig. 3D). With respect to liver repair, we determined PCNA expression in hepatocytes (Fig. 3E). WT → WT and WT → *TP*^−/−^ mice exhibited enhanced PCNA expression, while *TP*^−/−^ → WT and *TP*^−/−^ → *TP*^−/−^ mice exhibited reduced PCNA expression. We also determined the recruitment of macrophages to the liver. CD68^+^ cells in WT → WT and WT → *TP*^−/−^ mice were accumulated to the demarcated necrotic regions, while CD68^+^ cells in *TP*^−/−^ → WT and *TP*^−/−^ → *TP*^−/−^ mice were distributed to the entire liver lobules and were not concentrated in the damaged regions (Fig. 3F). The numbers of CD68^+^ cells in WT → WT and WT → *TP*^−/−^ mice were higher than those in *TP*^−/−^ → WT and *TP*^−/−^ → *TP*^−/−^ mice. Together, these results suggest that TP signaling in BM cells plays a critical role in the resolution of liver inflammation and repair in APAP-induced liver injury by accumulating macrophages in injured regions.

Taken together, these results indicated that the inhibition of TP signaling aggravated APAP-induced liver injury and that TP deficiency in BM cells enhanced liver injury and delayed liver repair after APAP treatment. Additionally, the impaired accumulation of macrophages in injured regions was associated with delayed liver repair following APAP-induced liver injury.

### Inhibition of TXS aggravated APAP-induced liver injury

We further confirmed that TP signaling is involved in liver repair during APAP hepatotoxicity. The TXS inhibitor Ozagrel or vehicle (PBS) was administered 2 h after APAP treatment, and liver injury and repair were assessed 48 h after APAP treatment. Ozagrel treatment sensitized mice to APAP-induced liver injury, as indicated by increased levels of ALT and hepatic necrotic area (Supplementary Fig. 4).

### Macrophage-specific TP deficiency exacerbated APAP-induced liver injury and delayed liver repair

**Fig. 4:**
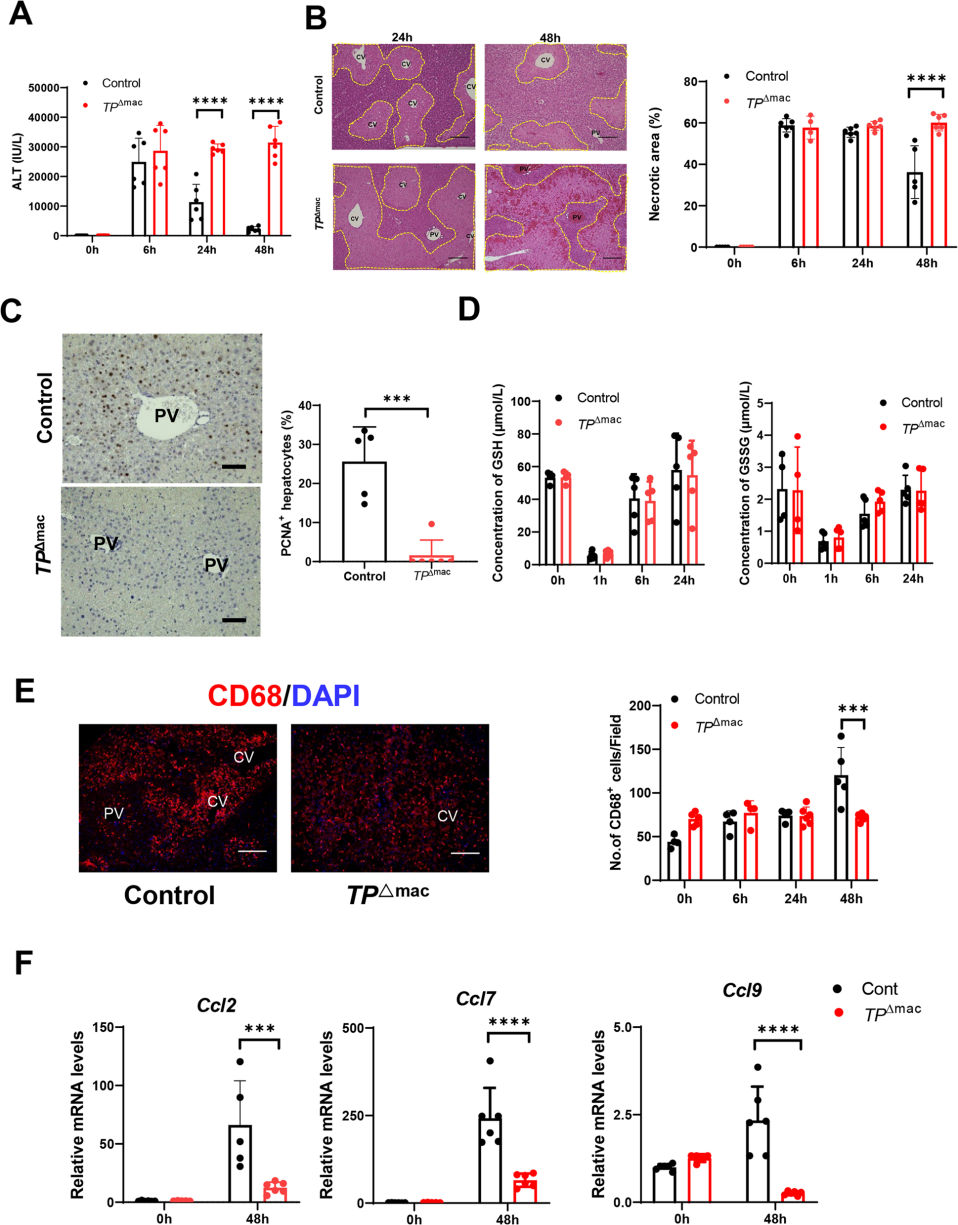
Deletion of TP signaling in macrophages delays liver repair after APAP treatment. **A** ALT levels after APAP treatment in Control and *TP*^△^^mac^ mice. Data are expressed as the mean ± SD (*n* = 6 mice per group). ***p < 0.001. **B** Representative photos of H&E staining of liver sections from Control and *TP*^△^^mac^ mice after APAP treatment. The yellow dotted lines indicate areas of necrosis. *CV* central vein, *PV* portal vein. Scale bars: 200 μm. Hepatic necrotic area (%) after APAP treatment. Data are expressed as the mean ± SD (*n* = 5–6 mice per group). ****p < 0.0001. **C** Representative images of PCNA immunostaining of liver sections from Control and *TP*^△^^mac^ mice 48 h after APAP treatment. *PV* portal vein. Scale bars: 100 μm. Percentage of PCNA^+^ hepatocytes 48 h after APAP treatment. Data are expressed as the mean ± SD (*n* = 5–6 mice per group). ***p < 0.001. **D** GSH and GSSG concentrations in the livers of Control and *TP*^△^^mac^ mice after APAP treatment. Data are expressed as the mean ± SD (*n* = 5–6 mice per group). **E** Immunofluorescence of CD68 (red) in the livers of Control and *TP*^△^^mac^ mice 48 h after APAP treatment. *CV* central vein, *PV* portal vein. Scale bars: 100 μm. The numbers of CD68^+^ macrophages in livers from Control and *TP*^△^^mac^ mice after APAP treatment. Data are expressed as the mean ± SD (*n* = 4–6 mice per group). ***p < 0.001. **F** Expression of genes encoding *Ccl2*, *Ccl7*, and *Ccl9* in the livers of control and *TP*^△^^mac^ mice 0 h and 48 h after APAP treatment. Data are expressed as the mean ± SD (*n* = 5–6 mice per group). ***p < 0.001; ****p < 0.0001

To confirm that TP signaling in macrophages plays a role in liver repair after APAP treatment, APAP was administered to macrophage-specific TP deficient (*TP*^△mac^) or control (Controls) mice. Compared with controls, *TP*^△mac^ mice exhibited sustained liver injury as indicated by increased levels of ALT (Fig. 4A) and necrotic area (Fig. 4B), suggesting that liver repair was delayed in *TP*^△mac^ mice. In addition, the expression of PCNA in *TP*^△mac^ mice was decreased at 48 h after APAP treatment as compared with controls (Fig. 4C).

Furthermore, changes in hepatic GSH levels, which detoxifies NAPQI via conjugation, were similar in the liver sections of both *TP*^△mac^ mice and controls (Fig. 4D). Both mouse models displayed rapid GSH depletion 1 h after APAP treatment, followed by gradual replenishment and recovery to pre-APAP levels within 24 h of APAP treatment. The same was true for the hepatic GSSG levels, which is a marker of oxidative stress. The similar rate of GSH depletion in the early phase of APAP hepatotoxicity between the two genotypes suggested that the difference in hepatotoxicity did not result from APAP metabolic activation.

To validate the pro-repairing role of TP signaling in macrophages, we employed another model of acute liver injury induced by concanavalin A (ConA). After administration of ConA to *TP*^△mac^ mice or controls, *TP*^△mac^ mice exhibited sustained liver injury, as indicated by increased levels of ALT and necrotic area and decreased PCNA expression in *TP*^△mac^ mice when compared with controls (Supplementary Fig. 5).

### Deficiency of TP signaling in macrophages suppressed accumulation of macrophages in the injured regions

To understand the contribution of TP signaling in macrophages to liver injury and repair following APAP treatment, we determined the number of macrophages. The numbers of CD68^+^ cells in controls was larger than that in *TP*^△mac^ mice at 48 h after APAP treatment (Fig. 4E). Immunofluorescence revealed that CD68^+^ cells accumulated extensively into the necrotic regions of livers from controls, whereas CD68^+^ cells diffusely distributed to the liver lobules from *TP*^△mac^ mice (Fig. 4E). These were accompanied by higher mRNA expression levels of *Ccl2*, *Ccl7*, and *Ccl9* in controls than those in *TP*^△mac^ mice (Fig. 4F).

### TP signaling regulates macrophage polarization and promotes liver repair

**Fig. 5:**
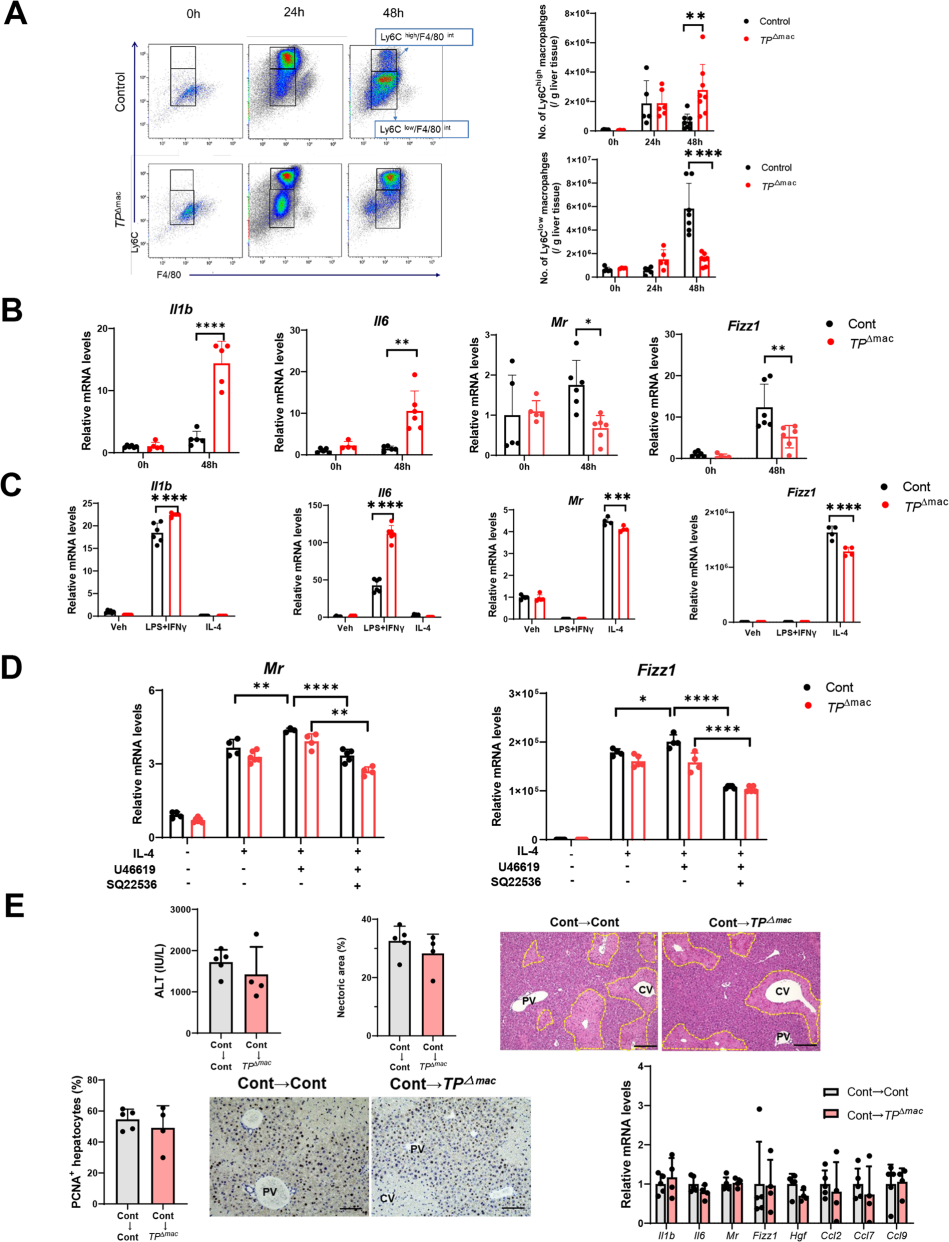
TP signaling in macrophages increases reparative macrophages and stimulates liver repair in *TP*^△^^mac^ mice. **A** Representative dot plots of liver pro-inflammatory and reparative macrophages in Control and *TP*^△^^mac^ mice 0 h, 24 h, and 48 h after APAP treatment. After gating out the Ly6G^+^ and CD11b^+^ cells, the cells were separated into two subsets based on the expression of Ly6C and F4/80. Ly6G^-^/Ly6C^high^/F4/80^int^ cells were defined as Ly6C^high^ macrophages (pro-inflammatory macrophages), and Ly6G^-^/Ly6C^low^/F4/80^i^^nt^ cells were defined as Ly6C^low^ macrophages (reparative macrophages). Changes in the numbers of Ly6C^high^ macrophages and Ly6C^low^ macrophages in livers from Control and *TP*^△^^mac^ mice after APAP treatment are demonstrated. Data are expressed as the mean ± SD (*n* = 5–6 mice per group). **p < 0.01; ****p < 0.0001. **B** Expression of mRNA encoding genes related to a pro-inflammatory macrophage phenotype (*Il1b and Il6*) and genes related to a reparative macrophage phenotype (*Mr *and* Fizz1*) in livers from Control and *TP*^△^^mac^ mice 0 h and 48 h after APAP treatment. Data are expressed as the mean ± SD (*n* = 5–6 mice per group). *p < 0.05; **p < 0.01; ****p < 0.0001. **C** Expression of mRNA encoding genes related to a pro-inflammatory macrophage phenotype (*Il1b and Il6*) and genes related to a reparative macrophage phenotype (*Mr *and* Fizz1*) in cultured BM-derived macrophages from Control and *TP*^△^^mac^ mice. BM-derived at day 7 of culture were stimulated with IFN-γ/LPS or IL-4 for 18 h. Data are expressed as the mean ± SD (*n* = 5–6 mice per group). ***p < 0.001; ****p < 0.0001. **D** Expression of mRNA encoding genes related to a reparative macrophage phenotype (*Mr *and* Fizz1*) in cultured BM-derived macrophages stimulated with IL-4 in the presence or absence of U46619 and SQ22536. Data are expressed as the mean ± SD (*n* = 3–6 mice per group). *p < 0.05; **p < 0.01; ****p < 0.0001. **E** Effects of adoptive transfer of BM-derived macrophages on APAP-induced liver injury. The ALT, hepatic necrotic area, and PCNA expression levels 48 h after APAP treatment in Control and *TP*^△^^mac^ mice transplanted with BM-derived macrophages from Control. BM-derived macrophages were transferred to mice 4 h after APAP administration. Representative photos of H&E and PCNA staining of liver sections. *CV* central vein, *PV* portal vein. Scale bars: 200 μm. the mRNA levels of pro-inflammatory (*Il1b *and* Il6) *and anti-inflammatory factors (*Mr* and *Fizz1*) and chemokines (*Ccl2*, *Ccl7*, and *Ccl9*) in the liver tissues 48 h after APAP treatment in Control and *TP*^△^^mac^ mice transplanted with BM-derived macrophages from Control. Data are expressed as the mean ± SD (*n* = 4–6 mice per group)

We further evaluated the phenotypes of infiltrated macrophages during APAP hepatotoxicity using flow cytometry. Flow cytometric analysis demonstrated that the populations of Ly6C^high^ macrophages (pro-inflammatory macrophages) in *TP*^△mac^ mice 48 h after APAP treatment were larger than those in control mice, while the populations of Ly6C^low^ macrophages (reparative macrophages) in controls were larger than those in *TP*^△mac^ mice (Fig. 5A). These results indicated that accelerated liver repair in controls was associated with accumulation of reparative macrophages and that delayed liver repair in *TP*^△mac^ mice was associated with accumulation of pro-inflammatory macrophages. We also determined the levels of pro- and anti-inflammatory mediators in the liver. The expressions of mRNA encoding pro-inflammatory mediators including *Il1b* and *Il6* in *TP*^△mac^ mice 48 h after APAP treatment were higher than those in controls (Fig. 5B). In addition, the expressions of mRNA encoding anti-inflammatory mediators including *Mr* and *Fizz1* in *TP*^△mac^ mice were lower than those in controls.

To further examine whether TP signaling in macrophages polarizes macrophages from a pro-inflammatory macrophage phenotype toward a reparative macrophage phenotype, BM-derived macrophages were stimulated using LPS/IFN-γ or IL-4. The administration of LPS/IFN-γ polarizes BM-macrophages toward a pro-inflammatory phenotype, whereas IL-4 administration polarizes BM-macrophages toward a reparative phenotype [22]. The levels of mRNA encoding *Il1b* and *Il6* in macrophages from *TP*^△mac^ mice stimulated with LPS/IFN-γ were enhanced as compared with those from controls (Fig. 5C). On the other hand, the mRNA expression levels of *Mr* and *Fizz1* were enhanced in macrophages from controls stimulated with IL-4 as compared with those from *TP*^△mac^ mice. These results suggest that TP signaling polarizes macrophages toward a reparative phenotype. To further determine whether the macrophage polarization is regulated through TP signaling, BM-derived macrophages from controls and *TP*^△mac^ mice were stimulated with IL-4 and the TP agonist, U46619. As shown in Fig. 5D, U46619 increased the mRNA levels of *Mr* and *Fizz1* in IL-4-treated macrophages from controls but not from *TP*^△mac^ mice, suggesting that macrophage polarization toward a reparative phenotype was dependent on TP signaling. Additionally, TXA_2_/TP signaling activates downstream pathways such as adenylyl cyclase (AC) [10], which is crucial for catalyzing the production of the second messenger, cyclic adenosine monophosphate (cAMP). cAMP has been shown to promote macrophage polarization toward a reparative phenotype [25]. To explore the involvement of the cAMP signaling pathway in TP signaling-mediated macrophage polarization, BM-macrophages were treated with the AC antagonist SQ22536. SQ22536 decreased the mRNA levels of *Mr* and *Fizz1* in controls and *TP*^△mac^ mouse macrophages treated with IL-4 and U46619. These results suggested that the activation of TP signaling promotes macrophage polarization through the cAMP pathway.

To verify the involvement of TP signaling in macrophages in liver repair from APAP-induced liver injury, BM-derived macrophages from controls were transferred to either controls or *TP*^△mac^ mice. The results showed that ALT, necrotic area, and PCNA levels 48 h after APAP treatment were comparable between the two groups (Fig. 5E). Additionally, the mRNA levels of pro-inflammatory and anti-inflammatory cytokines and chemokines did not show any significant differences. We also determined the effects of the adoptive transfer 24 h after APAP treatment. TP^△mac^ mice adoptively transferred with BM-derived macrophages from controls exhibited decreased levels of ALT and hepatic necrosis, but not CD68^+^ cells in the livers compared to *TP*^△mac^mice alone (Supplementary Fig. 6). These results indicated that TP-expressing macrophages rescued APAP-induced liver injury and facilitated liver repair in *TP*^△mac^ mice.

With respect to other immune cells, including Kuffer cells (KCs), the number of KCs in both genotypes decreased after APAP treatment (Supplementary Fig. 7). By contrast, neutrophils in controls transiently increased at 24 h, and the number of neutrophils at 24 h in controls was higher than that in *TP*^△mac^ mice. At 48 h, the number of neutrophils in controls declined, whereas that in *TP*^△mac^ mice was elevated. The number of neutrophils at 48 h in *TP*^△mac^ mice was higher than that in controls.

### HGF treatment attenuated APAP-induced liver injury and enhanced liver repair

**Fig. 6:**
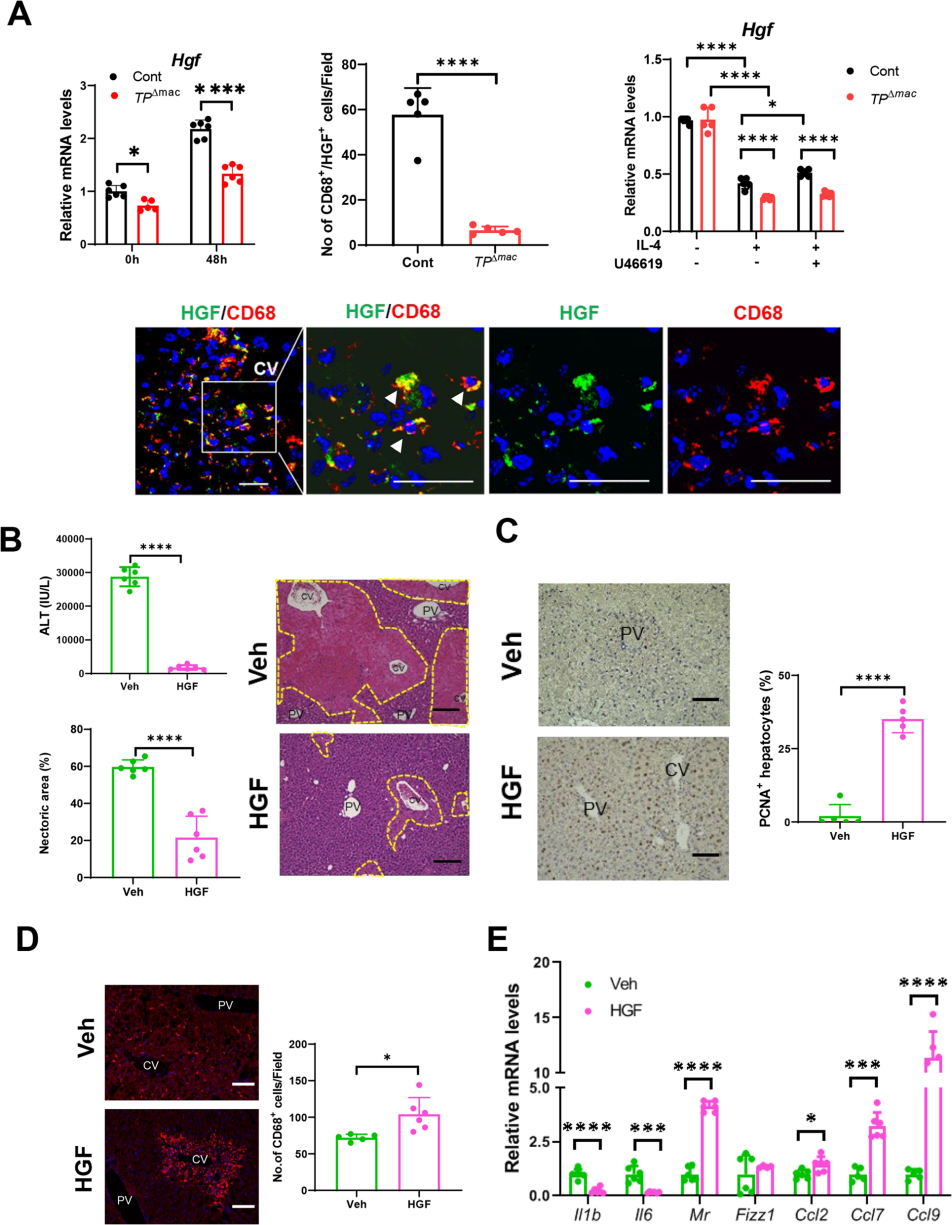
HGF administration reduced APAP-induced liver injury and facilitated liver repair. **A** Expression of mRNA encoding genes *Hgf* in the livers of *TP*^△^^mac^ or control mice 0 h and 48 h after APAP treatment. Data are expressed as the mean ± SD (*n* = 5–6 mice per group). Double immunofluorescence of HGF (green) and CD68 (red) in control livers at 48 h. The three right panels are magnified images of the area outlined by the white solid box in the left panel. Scale bar: 20 μm. Arrowheads indicate double-positive cells. Scale bar: 20 μm. The number of CD68^+^ cells positive for HGP in the livers of *TP*^△^^mac^ or Control mice 48 h after APAP treatment. Data are expressed as the mean ± SD (*n* = 5 mice per group). *p < 0.05; ****p < 0.0001. **B** ALT levels and hepatic necrotic area (%) in *TP*^△^^mac^ mice treated with HGF or vehicle at 48 h. Representative images of H&E staining of liver sections from *TP*^△^^mac^ mice treated with HGF or vehicle at 48 h. *CV* central vein, *PV* portal vein. Scale bars: 200 μm. **C** Representative images of PCNA staining of liver sections from *TP*^△^^mac^ mice treated with HGF or vehicle at 48 h. *CV* central vein, *PV* portal vein. Scale bars: 100 μm. PCNA^+^ hepatocytes (%) in *TP*^△^^mac^ mice treated with HGF or vehicle. **D** Representative immunofluorescence images of CD68 from *TP*^△^^mac^ mice treated with HGF or vehicle at 48 h. *CV* central vein, *PV* portal vein. Scale bars: 100 μm. CD68^+^ cells in *TP*^△^^mac^ mice treated with HGF or vehicle. **E** Expression of mRNA-encoding genes related to a pro-inflammatory macrophage phenotype (*Il1b *and* Il6*), genes related to a reparative macrophage phenotype (*Mr *and *Fizz1*), and genes related to chemokines (*Ccl2, Ccl7*, and *Ccl9*) in the livers of *TP*^△^^mac^ mice treated with HGF or vehicle 48 h after APAP treatment. Data are expressed as the mean ± SD. *p < 0.05; ***p < 0.001; ****p < 0.0001

HGF is critical for the protection and regeneration of liver tissues [26]. We previously reported that *TP*^−/−^ mice exhibited delayed liver repair after carbon tetrachloride-induced acute liver injury, which was associated with reduced HGF levels, suggesting that HGF contributes to liver repair [17, 18]. Considering these findings, to gain insight into the mechanisms by which TP signaling in macrophages facilitates liver repair after APAP-induced liver injury, we determined the hepatotrophic factor, HGF. mRNA levels of *Hgf* in *TP*^△mac^ mice were lower than those in controls (Fig. 6A). We also examined the localization of HGF. Immunofluorescence revealed that HGF colocalized with CD68^+^ cells in the injured centrilobular regions of controls 48 h after APAP treatment (Fig. 6A). The number of CD68^+^ cells expressing HGF in controls was higher than in *TP*^△mac^ mice. Additionally, the controls had a higher number of reparative macrophages than *TP*^△mac^ mice (Fig. 5A), suggesting that reparative macrophages expressing TP is responsible for producing HGF. To examine whether TP signaling in reparative macrophages stimulates HGF production, *Hgf* mRNA levels were determined in BM-derived macrophages treated with IL-4 in vitro (Fig. 6A). Although *Hgf* mRNA levels in IL-4-induced reparative macrophages from both genotypes were reduced, controls exhibited higher *Hgf* mRNA levels than *TP*^△mac^ mice. Additionally, TP stimulation by U46619 increased *Hgf* mRNA levels in controls but not in* TP*^△mac^ mice, suggesting that HGF was induced through TP signaling in macrophages.

To examine the contribution of HGF to promote liver repair, *TP*^△mac^ mice were given HGF 2 h and 24 h after APAP treatment. Treatment of *TP*^△mac^ mice with HGF attenuated APAP-induced liver injury as indicated by reduced levels of ALT and hepatic necrotic area (Fig. 6B) and enhanced liver repair as indicated by increased PCNA^+^ hepatocytes (Fig. 6C). The number of CD68^+^ cells in HGF-treated *TP*^△mac^ mice was higher than that in vehicle-treated *TP*^△mac^ mice (Fig. 6D). The levels of mRNA for *Il1b* and *Il6* in HGF-treated *TP*^△mac^ mice were lower than those in vehicle-treated *TP*^△mac^ mice, while the levels of mRNA for *Mr* in HGF-treated *TP*^△mac^ mice were higher than those in vehicle-treated *TP*^△mac^ mice (Fig. 6E). The mRNA levels of *Ccl2*, *Ccl7*, and *Ccl9* in HGF-treated *TP*^△mac^ mice were higher than those in vehicle-treated *TP*^△mac^ mice (Fig. 6E). These results suggest that HGF, probably from macrophages, reduced liver inflammation and facilitated liver repair after APAP treatment.

## Discussion

In the present study, we examined the role of endogenous TP signaling in liver repair following APAP-induced liver injury in mice. Our data showed that the inhibition of TP signaling or TXS aggravated APAP-induced liver inflammation and delayed liver repair following APAP-induced liver injury, which was related to the suppressed accumulation of macrophages in the injured regions. The deficiency of TP signaling in BM-derived macrophages suppresses the accumulation of macrophages in damaged areas during repair. In addition, the inhibition of TP signaling in macrophages prolonged liver inflammation and impaired liver repair, which was accompanied by reduced accumulation of reparative macrophages in the damaged area and downregulated expression of the hepatotrophic factor HGF. Treatment of mice deficient in TP signaling in macrophages with HGF mitigated liver inflammation and facilitated liver repair. These results suggest that the inhibition of TP signaling in macrophages delays liver repair after APAP hepatotoxicity by suppressing the accumulation of reparative macrophages and producing HGF.

The processes of liver repair and regeneration after acute liver injury determine the outcomes of patients with APAP hepatotoxicity [26, 27]. Although the mechanisms underlying liver repair appear to be complex [28, 29], macrophages are key players in liver repair after acute liver injury, including APAP hepatotoxicity [8]. Macrophage recruitment is a critical event in the resolution of liver inflammation and restoration of liver function. Our data revealed an extensive accumulation of macrophages in the hepatic necrotic area during the repair phase. The recruited macrophages were derived from the BM to remove necrotic debris and injured hepatocytes. The accumulation of macrophages in injured regions is a prerequisite for proper liver repair [22, 30, 31]. By contrast, macrophages were evenly distributed throughout the livers from *TP*^△mac^ mice and were not accumulated and concentrated in the damaged regions. These results indicate that the scattered distribution of macrophages is associated with impaired liver repair following APAP-induced acute liver injury. Furthermore, control mice had more infiltrated macrophages than *TP*^△mac^ mice, suggesting that accumulation of macrophages in the necrotic area would be required for adequate removal of necrotic tissues and consequently for liver recovery and regeneration [22, 32].

The importance of macrophages in liver repair after acute liver injury has been highlighted by the deletion of hepatic macrophages using clodronate liposomes [24]. Pre-elimination of KCs with clodronate liposomes exacerbated APAP-induced liver injury, which is consistent with the present results, demonstrating increased ALT, necrosis and reduced PCNA expression.

Macrophage recruitment to injured areas is driven by chemokine production CCL2 is important for the recruitment of MoMFs to promote liver repair following APAP toxicity [33]. Consistent with this, our data showed that higher levels of CCL2 were associated with macrophage accumulation and recovery from APAP-induced liver injury. Upregulation of CCL2 expression in macrophages is dependent on TP signaling. CCL2 is produced by injured hepatocytes in the centrilobular area and recruited by macrophages during APAP hepatotoxicity [34, 35]. In addition, deletion of C–C motif chemokine receptor 2 (CCR2), a receptor for CCL2, inhibits the accumulation of macrophages and clearance of necrotic cell debris in APAP-treated livers [35], suggesting that downregulated CCR2 expression might lead to the suppression of phagocytosis of necrotic tissues in APAP-treated livers. Furthermore, a recent study reported that other chemokines, including CCL7 and CCL9, were elevated during APAP-induced liver injury [36], which is consistent with our results. Our data demonstrated that CD68^+^ macrophage accumulation was reduced in TP-deficient mice, suggesting that TP signaling mediates macrophage recruitment. Because chimeric mice bearing TP-expressing BM cells had more accumulation of CD68^+^ macrophages than mice bearing TP-deficient BM cells, TP signaling regulates the recruitment of macrophages from the BM. Additionally, *TP*^△mac^ mice exhibited reductions in CD68^+^ macrophages and reparative macrophages as compared to controls, suggesting that TP signaling also regulates macrophage accumulation through differentiation of macrophages to a reparative macrophage phenotype.

Macrophages are instrumental in the repair and regeneration processes after acute liver damage [37]. Pro-inflammatory macrophages peak at 24 h after APAP treatment, and reparative macrophages peak at 48 h [9], suggesting in situ reprogramming of the macrophage phenotype [38, 39]. The current study showed that increased numbers of reparative macrophages were associated with the promotion of liver repair, whereas increased numbers of pro-inflammatory macrophages were associated with the impairment of liver repair. These changes were associated with increased expression of genes related to the pro-inflammatory macrophage phenotype and decreased expression of genes related to the reparative macrophage phenotype in the livers of mice deficient in TP signaling. These results suggest that TP signaling in macrophages is involved in macrophage differentiation to repair damaged liver tissues exposed to APAP overdoses. Additionally, TP signaling in macrophages appears to be involved in macrophage polarization [15]. In vitro studies have demonstrated that the TP agonist, U46619, increased the expression of genes related to the reparative macrophage phenotype in IL-4-treated macrophages from controls, but not *TP*^△mac^ mice, suggesting that macrophage polarization depends on TP signaling. Additionally, the AC inhibitor, SQ22536 decreased the expression of genes related to the reparative macrophage phenotype in macrophages stimulated with IL-4 and U46619, suggesting that TP signaling promotes macrophage polarization toward a reparative phenotype through the cAMP pathway. AC-mediated activation of cAMP has been shown to promote macrophage polarization toward a reparative phenotype [25]. However, recent findings suggest that TP signaling leads to the polarization of adipose tissue macrophages toward a pro-inflammatory phenotype through protein kinase C activation [40]. These discrepant results may be attributed to the different experimental protocols employed.

Cells other than MoMFs are likely involved in liver repair following APAP hepatotoxicity [41]. The current study demonstrated that APAP treatment reduced the number of liver-resident macrophages and KCs in both genotypes. Loss of KCs is a common feature of liver diseases [42] including APAP toxicity [9, 30, 36]. These results suggest that KCs contribute little to liver repair via TP signaling in macrophages. Neutrophils are another type of immune cells that accumulate in APAP-treated livers. However, the role of neutrophils in the APAP-induced liver injury and repair remains controversial [43]. The present study demonstrated that control mice had more hepatic neutrophils at 24 h and a smaller hepatic necrotic area at 48 h, whereas mice lacking TP in macrophages had more hepatic neutrophils and more robust hepatic necrosis at 48 h. These results suggest that the accumulation of neutrophils prior to the accumulation of reparative macrophages may induce liver repair, and excessive accumulation of neutrophils in sustained inflammatory livers impairs the resolution of inflammation and delays liver repair [44]. Thus, the role of neutrophil accumulation in APAP hepatotoxicity appears to be context-dependent. Additionally, it remains unclear why the deletion of TP signaling in macrophages prevented the accumulation of neutrophils in the injured regions induced by APAP treatment. Further studies are necessary to elucidate the underlying mechanisms that regulate the role of neutrophils in APAP hepatotoxicity and their involvement in TP signaling.

Hepatic accumulation of platelets is also involved in the progression of APAP-induced liver injury and delays liver repair after APAP treatment [45, 46]. Because platelets aggregate through TP signaling, TP signaling in platelets would participate in APAP hepatotoxicity; however, in the present study, the area of platelet did not differ between WT and *TP*^−/−^ mice, suggesting that TP signaling-induced platelet accumulation did not contribute to APAP-induced liver injury and repair. These results indicate that platelet accumulation and aggregation in the liver are independent of TP signaling.

Various trophic factors facilitate liver regeneration and repair after APAP toxicity [26, 29]. We found that hepatic HGF levels in control mice were higher than those in *TP*^△mac^ mice 48 h after APAP treatment, which was consistent with our previous results [30]. Furthermore, the current study demonstrated that HGF administration attenuated APAP-induced liver injury and facilitated liver repair from the injury. HGF is characterized by pro-proliferative properties. HGF deletion impairs liver regeneration in mice [47]. In contrast, recombinant HGF appears to confer protection against liver failure by boosting liver regeneration in mice [48]. Consistent with this, HGF overexpression in BM-derived mesenchymal stem cells facilitates liver repair following liver injury [49]. In addition, transplantation of HGF-knockout mesenchymal stem cells reduced the survival rate of APAP-treated mice, and HGF administration increased the survival rate during APAP hepatotoxicity, indicating that HGF improved mouse liver failure induced by APAP administration.

Macrophages are one of the main sources of HGF during liver repair and regeneration. Our data showed that macrophages expressing HGF displayed increased accumulation in the livers of control mice compared to *TP*^△mac^ mice. Because control mice had a higher number of reparative macrophages compared to *TP*^△mac^ mice, reparative macrophages expressing TP would produce HGF. This view is supported by the observation that, in liver regeneration induced by partial hepatectomy in mice, accumulated reparative macrophages generate HGF, which contributes to hepatocyte proliferation [50]. Further studies are necessary to elucidate the role of HGF in macrophages for liver repair and regeneration from APAP-induced liver injury. Additionally, the current study showed that although *Hgf* mRNA levels were lower in reparative macrophages than in non-polarized macrophages, reparative macrophages increased *Hgf* expression through TP signaling. Further studies are warranted to explore the underlying molecular mechanism by which reparative macrophages produce HGF through TP signaling. The other possible origins of HGF secretion during APAP hepatotoxicity are likely located in hepatic stellate cells (HSCs) and liver sinusoidal endothelial cells (LSECs) [36].

In conclusion, our study demonstrates that TP signaling in macrophages facilitates liver repair after APAP overdose. Accumulation of TP-expressing macrophages in injured regions induced by APAP is a critical step in the resolution of liver inflammation and recovery. The activation of TP signaling in macrophages may be a therapeutic target for APAP-induced liver injury and liver recovery. Specific activation of TP signaling in macrophages may be a therapeutic target for liver repair and regeneration after APAP hepatotoxicity.

## Supplementary Information

Additional file 1: Supplementary Figure 1. Accumulation of CD41^+^ cells (platelets) in the livers after APAP treatment. (A) Representative photos showing immunofluorescence staining of CD41 (red) in the livers from WT and *TP*^−/−^ mice 0 h and 48 h after APAP treatment. CV, central vein. Scale bars: 100 μm. (B) The percentage of CD41^+^ cells in the livers from WT and TP^−/−^ mice after APAP treatment. Data are expressed as the mean ± SD (*n* = 3–5 mice per group). Supplementary Figure 2. CD68^+^ macrophages expressing TP in WT livers during APAP hepatotoxicity. Representative microphotographs of double-immunofluorescence staining for TP (green) and CD68 (red) in the livers from WT mice after APAP treatment. Arrowheads indicate merged cells. CV, central vein. Scale bars: 50 μm. Supplementary Figure 3. Clodronate liposomes (CL) reduced the number of macrophages after APAP treatment. Representative dot plots of liver macrophages (F4/80^+^/CD11b^+^ cells) gated out CD45^+^/Ly6G^high^/CD11b^high^ cells and the number of macrophages 48 h after APAP treatment in WT mice treated with control liposomes (Cont) and CL. Data are expressed as the mean ± SD (*n* = 4-6 mice per group). **p < 0.01. Supplementary Figure 4. The TXS inhibitor Ozagrel aggravated APAP-induced liver injury. The TXS inhibitor Ozagrel increased the levels of ALT and hepatic necrotic area 48 h after APAP treatment as compared with vehicle. Representative photos of H&E staining of liver sections. CV, central vein. PV, portal vein. Scale bars: 200 μm. Data are expressed as the mean ± SD (*n* = 4–6 mice per group).**p < 0.01, ***p < 0.001. Supplementary Figure 5. Delayed liver repair after concanavalin A (ConA)-induced liver injury in *TP*^△^^mac^ mice. The levels of ALT and hepatic necrotic area were increased and PCNA expression was decreased 48 h after concanavalin A (ConA) treatment in *TP*^△^^mac^ mice as compared with Controls. Representative images of H&E and PCNA staining of liver sections. CV, central vein. PV, portal vein. Scale bars: 200 μm. Data are expressed as the mean ± SD (*n* = 4–6 mice per group). * p< 0.05, **p < 0.01, ****p < 0.0001. Supplementary Figure 6. Effects of the adoptive transfer of BM-derived macrophages on APAP-induced liver injury. ALT levels, hepatic necrotic area, and CD68+ cells 24 h after APAP treatment in *TP*^△^^mac^ mice with and without the transfer of BM-derived macrophages from Control. BM-derived macrophages were transferred to mice 4 h after APAP administration. Representative photos of H&E and CD68 staining of liver sections. CV, central vein. PV, portal vein. Scale bars: 200 μm. Data are expressed as the mean ± SD (*n* = 5 mice per group). **p < 0.01, ****p< 0.0001. Supplementary Figure 7. Changes in Kupffer cells and neutrophils in the livers after APAP treatment. (A) Flow cytometry gating strategy used to identify Kupffer cells and neutrophils in livers from WT mice 0 h after APAP treatment. (B) Changes in the numbers of Kupffer cells and neutrophils after APAP treatment. Neutrophils were defined as Ly6G^+^ and CD11b^+^ cells, and Kupffer cells were defined as Ly6G^-^/ CD11b^int^ /Ly6C^low^/F4/80^high^-cells. Data are expressed as the mean ± SD (*n* = 4–6 mice per group). * p < 0.05, **p < 0.01, ****p < 0.0001. Supplementary Table 1. The primers used for reverse transcription and quantitative PCR reactions.

## Data Availability

All data generated or analyzed during this study are included within the article.

## Abbreviations

AC: Adenylate cyclase
ALT: Alanine transaminase
APAP: *N*-Acetyl-p-aminophenol
BM: Bone marrow
cAMP: Cyclic adenosine monophosphate
CCR2: C-C motif chemokine receptor 2
CL: Clodronate liposomes
DAPI: 4′-6-Diamidino-2-phenylindole
HGF: Hepatocyte growth factor
GFP: Green fluorescent protein
GSH: Glutathione
GSSG: Glutathione disulfide
H&E: Hematoxylin and eosin
IFN-γ: Interferon-gamma
IL: Interleukin
KC: Kupffer cell
LPS: Lipopolysaccharide
MoMF: Monocyte-derived macrophages
NAPQI: *N*-Acetyl-p-benzoquinone imine
PBS: Phosphate-buffered saline
PCNA: Proliferating cell nuclear antigen
TP: Thromboxane prostanoid
TXA_2_: Thromboxane A_2_
TXS: Thromboxane synthase
WT: Wild type

## References

[1] Stravitz RT, Fontana RJ, Karvellas C, Durkalski V, McGuire B, Rule JA, et al. Future directions in acute liver failure. Hepatology. 2023;78(4):1266–89. 10.1097/HEP.0000000000000458. 37183883 10.1097/HEP.0000000000000458 PMC10521792

[2] Ramachandran A, Jaeschke H. A mitochondrial journey through acetaminophen hepatotoxicity. Food Chem Toxicol. 2020;140:111282. 10.1016/j.fct.2020.111282. 32209353 10.1016/j.fct.2020.111282 PMC7254872

[3] Kubes P, Mehal WZ. Sterile inflammation in the liver. Gastroenterology. 2012;143(5):1158–72. 10.1053/j.gastro.2012.09.008. 22982943 10.1053/j.gastro.2012.09.008

[4] Mossanen JC, Krenkel O, Ergen C, Govaere O, Liepelt A, Puengel T, et al. Chemokine (C-C motif) receptor 2-positive monocytes aggravate the early phase of acetaminophen-induced acute liver injury. Hepatology. 2016;64(5):1667–82. 10.1002/hep.28682. 27302828 10.1002/hep.28682

[5] Heymann F, Mossanen JC, Peiseler M, Niemietz PM, Araujo David B, Krenkel O, et al. Hepatic C-X-C chemokine receptor type 6-expressing innate lymphocytes limit detrimental myeloid hyperactivation in acute liver injury. Hepatol Commun. 2023;7(4):e0102. 10.1097/HC9.0000000000000102. 36972392 10.1097/HC9.0000000000000102 PMC10503691

[6] Wynn TA, Vannella KM. Macrophages in tissue repair, regeneration, and fibrosis. Immunity. 2016;44(3):450–62. 10.1016/j.immuni.2016.02.015. 26982353 10.1016/j.immuni.2016.02.015 PMC4794754

[7] David BA, Rezende RM, Antunes MM, Santos MM, Freitas Lopes MA, Diniz AB, et al. Combination of mass cytometry and imaging analysis reveals origin, location, and functional repopulation of liver myeloid cells in mice. Gastroenterology. 2016;151(6):1176–91. 10.1053/j.gastro.2016.08.024. 27569723 10.1053/j.gastro.2016.08.024

[8] Krenkel O, Tacke F. Liver macrophages in tissue homeostasis and disease. Nat Rev Immunol. 2017;17(5):306–21. 10.1038/nri.2017.11. 28317925 10.1038/nri.2017.11

[9] Zigmond E, Samia-Grinberg S, Pasmanik-Chor M, Brazowski E, Shibolet O, Halpern Z, et al. Infiltrating monocyte-derived macrophages and resident kupffer cells display different ontogeny and functions in acute liver injury. J Immunol. 2014;193(1):344–53. 10.4049/jimmunol.1400574. 24890723 10.4049/jimmunol.1400574

[10] Narumiya S, Sugimoto Y, Ushikubi F. Prostanoid receptors: structures, properties, and functions. Physiol Rev. 1999;79(4):1193–226. 10.1152/physrev.1999.79.4.1193. 10508233 10.1152/physrev.1999.79.4.1193

[11] Katagiri H, Ito Y, Ishii K, Hayashi I, Suematsu M, Yamashina S, et al. Role of thromboxane derived from COX-1 and -2 in hepatic microcirculatory dysfunction during endotoxemia in mice. Hepatology. 2004;39(1):139–50. 10.1002/hep.20000. 14752832 10.1002/hep.20000

[12] Teoh NC, Ito Y, Field J, Bethea NW, Amr D, McCuskey MK, et al. Diannexin, a novel annexin V homodimer, provides prolonged protection against hepatic ischemia-reperfusion injury in mice. Gastroenterology. 2007;133(2):632–46. 10.1053/j.gastro.2007.05.027. 17681182 10.1053/j.gastro.2007.05.027

[13] Yamane S, Amano H, Ito Y, Betto T, Matsui Y, Koizumi W, et al. The role of thromboxane prostanoid receptor signaling in gastric ulcer healing. Int J Exp Pathol. 2022;103(1):4–12. 10.1111/iep.12410. 34655121 10.1111/iep.12410 PMC8781669

[14] Amano H, Ito Y, Eshima K, Kato S, Ogawa F, Hosono K, et al. Thromboxane A2 induces blood flow recovery via platelet adhesion to ischaemic regions. Cardiovasc Res. 2015;107(4):509–21. 10.1093/cvr/cvv139. 25935870 10.1093/cvr/cvv139

[15] Mishima T, Hosono K, Tanabe M, Ito Y, Majima M, Narumiya S, et al. Thromboxane prostanoid signaling in macrophages attenuates lymphedema and facilitates lymphangiogenesis in mice: TP signaling and lymphangiogenesis. Mol Biol Rep. 2023;50(10):7981–93. 10.1007/s11033-023-08620-0. 37540456 10.1007/s11033-023-08620-0 PMC10520203

[16] Matsuda H, Ito Y, Hosono K, Tsuru S, Inoue T, Nakamoto S, et al. Roles of Thromboxane receptor signaling in enhancement of lipopolysaccharide-induced lymphangiogenesis and lymphatic drainage function in diaphragm. Arterioscler Thromb Vasc Biol. 2021;41(4):1390–407. 10.1161/ATVBAHA.120.315507. 33567865 10.1161/ATVBAHA.120.315507

[17] Minamino T, Ito Y, Ohkubo H, Hosono K, Suzuki T, Sato T, et al. Thromboxane A(2) receptor signaling promotes liver tissue repair after toxic injury through the enhancement of macrophage recruitment. Toxicol Appl Pharmacol. 2012;259(1):104–14. 10.1016/j.taap.2011.12.013. 22206755 10.1016/j.taap.2011.12.013

[18] Minamino T, Ito Y, Ohkubo H, Shimuzu Y, Kojo K, Nishizwa N, et al. Adhesion of platelets through thromboxane A(2) receptor signaling facilitates liver repair during acute chemical-induced hepatotoxicity. Life Sci. 2015;132:85–92. 10.1016/j.lfs.2015.03.015. 25921763 10.1016/j.lfs.2015.03.015

[19] Takayama K, Yuhki K, Ono K, Fujino T, Hara A, Yamada T, et al. Thromboxane A2 and prostaglandin F2alpha mediate inflammatory tachycardia. Nat Med. 2005;11(5):562–6. 10.1038/nm1231. 15834430 10.1038/nm1231

[20] Vincenti F, Kim J, Gouveia D, Pelle G, Mayne TJ, Neylan JF. Phase 3 trial design of the hepatocyte growth factor mimetic ANG-3777 in renal transplant recipients with delayed graft function. Kidney Int Rep. 2021;6(2):296–303. 10.1016/j.ekir.2020.11.001. 33615054 10.1016/j.ekir.2020.11.001 PMC7879201

[21] Inoue T, Ito Y, Nishizawa N, Eshima K, Kojo K, Otaka F, et al. RAMP1 in Kupffer cells is a critical regulator in immune-mediated hepatitis. PLoS ONE. 2018;13(11):e0200432. 10.1371/journal.pone.0200432. 30462657 10.1371/journal.pone.0200432 PMC6248891

[22] Nishizawa N, Ito Y, Eshima K, Ohkubo H, Kojo K, Inoue T, et al. Inhibition of microsomal prostaglandin E synthase-1 facilitates liver repair after hepatic injury in mice. J Hepatol. 2018;69(1):110–20. 10.1016/j.jhep.2018.02.009. 29458169 10.1016/j.jhep.2018.02.009

[23] Hsia CW, Shu LH, Lee AW, Tran OT, Yang CH, Yen TL, et al. Ginkgetin effectively mitigates collagen and AA-induced platelet activation via PLCgamma2 but not cyclic nucleotide-dependent pathway in human. J Cell Mol Med. 2024;28(4):e18139. 10.1111/jcmm.18139. 38334198 10.1111/jcmm.18139 PMC10853947

[24] Ju C, Reilly TP, Bourdi M, Radonovich MF, Brady JN, George JW, et al. Protective role of Kupffer cells in acetaminophen-induced hepatic injury in mice. Chem Res Toxicol. 2002;15(12):1504–13. 10.1021/tx0255976. 12482232 10.1021/tx0255976

[25] Polumuri S, Perkins DJ, Vogel SN. cAMP levels regulate macrophage alternative activation marker expression. Innate Immun. 2021;27(2):133–42. 10.1177/1753425920975082. 33241977 10.1177/1753425920975082 PMC7882807

[26] Michalopoulos GK, Bhushan B. Liver regeneration: biological and pathological mechanisms and implications. Nat Rev Gastroenterol Hepatol. 2021;18(1):40–55. 10.1038/s41575-020-0342-4. 32764740 10.1038/s41575-020-0342-4

[27] Mehendale HM. Tissue repair: an important determinant of final outcome of toxicant-induced injury. Toxicol Pathol. 2005;33(1):41–51. 10.1080/01926230590881808. 15805055 10.1080/01926230590881808

[28] Jaeschke H, Williams CD, Ramachandran A, Bajt ML. Acetaminophen hepatotoxicity and repair: the role of sterile inflammation and innate immunity. Liver Int. 2012;32(1):8–20. 10.1111/j.1478-3231.2011.02501.x. 21745276 10.1111/j.1478-3231.2011.02501.x PMC3586825

[29] Bhushan B, Apte U. Liver regeneration after acetaminophen hepatotoxicity: mechanisms and therapeutic opportunities. Am J Pathol. 2019;189(4):719–29. 10.1016/j.ajpath.2018.12.006. 30653954 10.1016/j.ajpath.2018.12.006 PMC6446224

[30] Kato T, Ito Y, Hosono K, Suzuki T, Tamaki H, Minamino T, et al. Vascular endothelial growth factor receptor-1 signaling promotes liver repair through restoration of liver microvasculature after acetaminophen hepatotoxicity. Toxicol Sci. 2011;120(1):218–29. 10.1093/toxsci/kfq366. 21135413 10.1093/toxsci/kfq366

[31] Nakamoto S, Ito Y, Nishizawa N, Goto T, Kojo K, Kumamoto Y, et al. Lymphangiogenesis and accumulation of reparative macrophages contribute to liver repair after hepatic ischemia-reperfusion injury. Angiogenesis. 2020;23(3):395–410. 10.1007/s10456-020-09718-w. 32162023 10.1007/s10456-020-09718-w

[32] Feng D, Xiang X, Guan Y, Guillot A, Lu H, Chang C, et al. Monocyte-derived macrophages orchestrate multiple cell-type interactions to repair necrotic liver lesions in disease models. J Clin Invest. 2023;133(15):e166954. 10.1172/JCI166954. 37338984 10.1172/JCI166954 PMC10378165

[33] Holt MP, Cheng L, Ju C. Identification and characterization of infiltrating macrophages in acetaminophen-induced liver injury. J Leukoc Biol. 2008;84(6):1410–21. 10.1189/jlb.0308173. 18713872 10.1189/jlb.0308173 PMC2614594

[34] Antoniades CG, Quaglia A, Taams LS, Mitry RR, Hussain M, Abeles R, et al. Source and characterization of hepatic macrophages in acetaminophen-induced acute liver failure in humans. Hepatology. 2012;56(2):735–46. 10.1002/hep.25657. 22334567 10.1002/hep.25657

[35] Dambach DM, Watson LM, Gray KR, Durham SK, Laskin DL. Role of CCR2 in macrophage migration into the liver during acetaminophen-induced hepatotoxicity in the mouse. Hepatology. 2002;35(5):1093–103. 10.1053/jhep.2002.33162. 11981759 10.1053/jhep.2002.33162

[36] Ben-Moshe S, Veg T, Manco R, Dan S, Papinutti D, Lifshitz A, et al. The spatiotemporal program of zonal liver regeneration following acute injury. Cell Stem Cell. 2022;29(6):973–89 e10. 10.1016/j.stem.2022.04.008. 35659879 10.1016/j.stem.2022.04.008

[37] Kolodziejczyk AA, Federici S, Zmora N, Mohapatra G, Dori-Bachash M, Hornstein S, et al. Acute liver failure is regulated by MYC- and microbiome-dependent programs. Nat Med. 2020;26(12):1899–911. 10.1038/s41591-020-1102-2. 33106666 10.1038/s41591-020-1102-2

[38] Dal-Secco D, Wang J, Zeng Z, Kolaczkowska E, Wong CH, Petri B, et al. A dynamic spectrum of monocytes arising from the in situ reprogramming of CCR2+ monocytes at a site of sterile injury. J Exp Med. 2015;212(4):447–56. 10.1084/jem.20141539. 25800956 10.1084/jem.20141539 PMC4387291

[39] Ramachandran P, Pellicoro A, Vernon MA, Boulter L, Aucott RL, Ali A, et al. Differential Ly-6C expression identifies the recruited macrophage phenotype, which orchestrates the regression of murine liver fibrosis. Proc Natl Acad Sci U S A. 2012;109(46):E3186–95. 10.1073/pnas.1119964109. 23100531 10.1073/pnas.1119964109 PMC3503234

[40] Xu R, Dai Y, Zheng X, Yan Y, He Z, Zhang H, et al. Thromboxane A(2)-TP axis promotes adipose tissue macrophages M1 polarization leading to insulin resistance in obesity. Biochem Pharmacol. 2023;210:115465. 10.1016/j.bcp.2023.115465. 36849064 10.1016/j.bcp.2023.115465

[41] Jaeschke H, Ramachandran A. Acetaminophen hepatotoxicity: paradigm for understanding mechanisms of drug-induced liver injury. Annu Rev Pathol. 2024;19:453–78. 10.1146/annurev-pathmechdis-051122-094016. 38265880 10.1146/annurev-pathmechdis-051122-094016 PMC11131139

[42] Ito Y, Hosono K, Amano H. Responses of hepatic sinusoidal cells to liver ischemia-reperfusion injury. Front Cell Dev Biol. 2023;11:1171317. 10.3389/fcell.2023.1171317. 37082623 10.3389/fcell.2023.1171317 PMC10112669

[43] Jaeschke H, Ramachandran A. Mechanisms and pathophysiological significance of sterile inflammation during acetaminophen hepatotoxicity. Food Chem Toxicol. 2020;138:111240. 10.1016/j.fct.2020.111240. 32145352 10.1016/j.fct.2020.111240 PMC7098420

[44] Kojo K, Ito Y, Eshima K, Nishizawa N, Ohkubo H, Yokomizo T, et al. BLT1 signalling protects the liver against acetaminophen hepatotoxicity by preventing excessive accumulation of hepatic neutrophils. Sci Rep. 2016;6:29650. 10.1038/srep29650. 27404729 10.1038/srep29650 PMC4939602

[45] Miyakawa K, Joshi N, Sullivan BP, Albee R, Brandenberger C, Jaeschke H, et al. Platelets and protease-activated receptor-4 contribute to acetaminophen-induced liver injury in mice. Blood. 2015;126(15):1835–43. 10.1182/blood-2014-09-598656. 26179083 10.1182/blood-2014-09-598656 PMC4600019

[46] Chauhan A, Sheriff L, Hussain MT, Webb GJ, Patten DA, Shepherd EL, et al. The platelet receptor CLEC-2 blocks neutrophil mediated hepatic recovery in acetaminophen induced acute liver failure. Nat Commun. 2020;11(1):1939. 10.1038/s41467-020-15584-3. 32321925 10.1038/s41467-020-15584-3 PMC7176690

[47] Phaneuf D, Moscioni AD, LeClair C, Raper SE, Wilson JM. Generation of a mouse expressing a conditional knockout of the hepatocyte growth factor gene: demonstration of impaired liver regeneration. DNA Cell Biol. 2004;23(9):592–603. 10.1089/dna.2004.23.592. 15383179 10.1089/dna.2004.23.592

[48] Kosai K, Matsumoto K, Funakoshi H, Nakamura T. Hepatocyte growth factor prevents endotoxin-induced lethal hepatic failure in mice. Hepatology. 1999;30(1):151–9. 10.1002/hep.510300102. 10385651 10.1002/hep.510300102

[49] Zhang Y, Li R, Rong W, Han M, Cui C, Feng Z, et al. Therapeutic effect of hepatocyte growth factor-overexpressing bone marrow-derived mesenchymal stem cells on CCl(4)-induced hepatocirrhosis. Cell Death Dis. 2018;9(12):1186. 10.1038/s41419-018-1239-9. 30538216 10.1038/s41419-018-1239-9 PMC6290007

[50] Huang M, Jiao J, Cai H, Zhang Y, Xia Y, Lin J, et al. C-C motif chemokine ligand 5 confines liver regeneration by down-regulating reparative macrophage-derived hepatocyte growth factor in a forkhead box O 3a-dependent manner. Hepatology. 2022;76(6):1706–22. 10.1002/hep.32458. 35288960 10.1002/hep.32458 PMC9790589

