# Supplementary material for: Deletion of TP signaling in macrophages delays liver repair following APAP-induced liver injury by reducing accumulation of reparative macrophage and production of HGF
Source: Inflamm Regen. 2024 Oct 3;44:43. doi: 10.1186/s41232-024-00356-z (PMC11451145; doi:10.1186/s41232-024-00356-z)
Supplement: Supplementary file 1 — Additional file 1: Supplementary Figure 1. Accumulation of CD41+ cells (platelets) in the livers after APAP treatment. (A) Representative photos showing immunofluorescence staining of CD41 (red) in the livers from WT and TP−/− mice 0 h and 48 h after APAP treatment. CV, central vein. Scale bars: 100 μm. (B) The percentage of CD41+ cells in the livers from WT and TP−/− mice after APAP treatment. Data are expressed as the mean ± SD (n = 3–5 mice per group). Supplementary Figure 2. CD68+ macrophages expressing TP in WT livers during APAP hepatotoxicity. Representative microphotographs of double-immunofluorescence staining for TP (green) and CD68 (red) in the livers from WT mice after APAP treatment. Arrowheads indicate merged cells. CV, central vein. Scale bars: 50 μm. Supplementary Figure 3. Clodronate liposomes (CL) reduced the number of macrophages after APAP treatment. Representative dot plots of liver macrophages (F4/80+/CD11b+ cells) gated out CD45+/Ly6Ghigh/CD11bhigh cells and the number of macrophages 48 h after APAP treatment in WT mice treated with control liposomes (Cont) and CL. Data are expressed as the mean ± SD (n = 4-6 mice per group). **p < 0.01. Supplementary Figure 4. The TXS inhibitor Ozagrel aggravated APAP-induced liver injury. The TXS inhibitor Ozagrel increased the levels of ALT and hepatic necrotic area 48 h after APAP treatment as compared with vehicle. Representative photos of H&E staining of liver sections. CV, central vein. PV, portal vein. Scale bars: 200 μm. Data are expressed as the mean ± SD (n = 4–6 mice per group).**p < 0.01, ***p < 0.001. Supplementary Figure 5. Delayed liver repair after concanavalin A (ConA)-induced liver injury in TP△mac mice. The levels of ALT and hepatic necrotic area were increased and PCNA expression was decreased 48 h after concanavalin A (ConA) treatment in TP△mac mice as compared with Controls. Representative images of H&E and PCNA staining of liver sections. CV, central vein. PV, portal vein. Scale bars: 2 [file 41232_2024_356_MOESM1_ESM.docx]

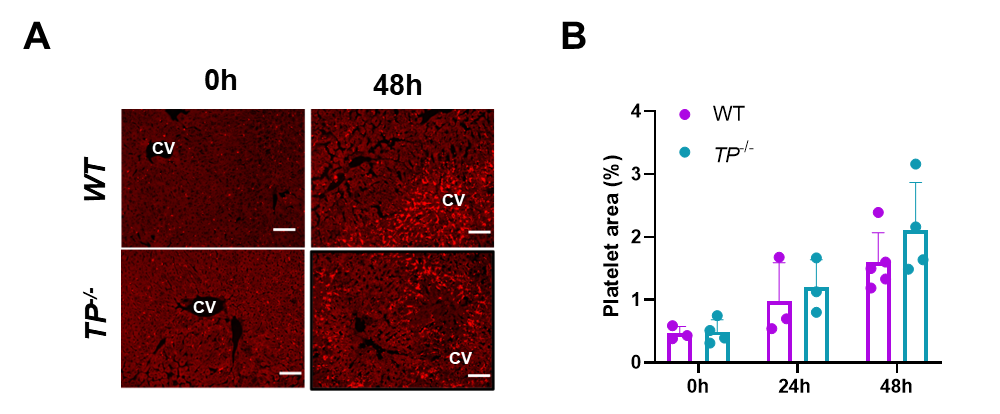


Supplementary Figure 1.

Accumulation of CD41^+^ cells (platelets) in the livers after APAP treatment.

(A) Representative photos showing immunofluorescence staining of CD41 (red) in the livers from WT and *TP*^−/−^ mice 0 h and 48 h after APAP treatment. CV, central vein. Scale bars: 100 μm. (B) The percentage of CD41^+^ cells in the livers from WT and TP^−/−^ mice after APAP treatment. Data are expressed as the mean ± SD (n = 3–5 mice per group).


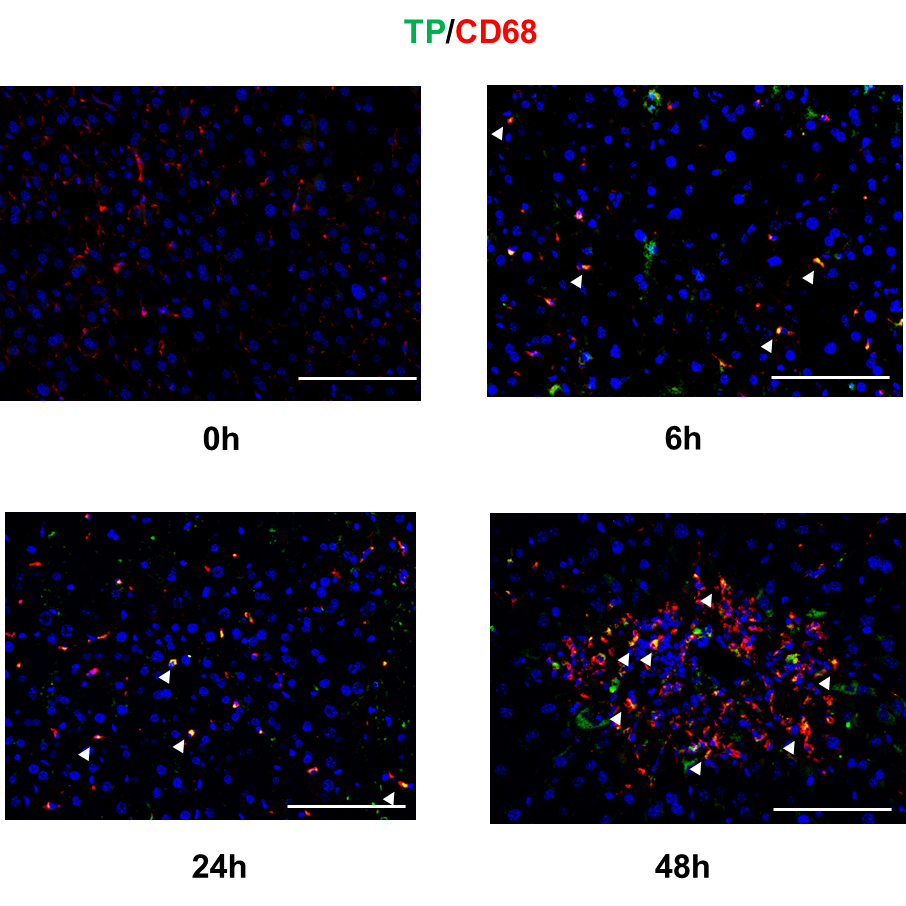


Supplementary Figure 2

CD68^+^ macrophages expressing TP in WT livers during APAP hepatotoxicity.

Representative microphotographs of double-immunofluorescence staining for TP (green) and CD68 (red) in the livers from WT mice after APAP treatment. Arrowheads indicate merged cells. CV, central vein. Scale bars: 50 μm.


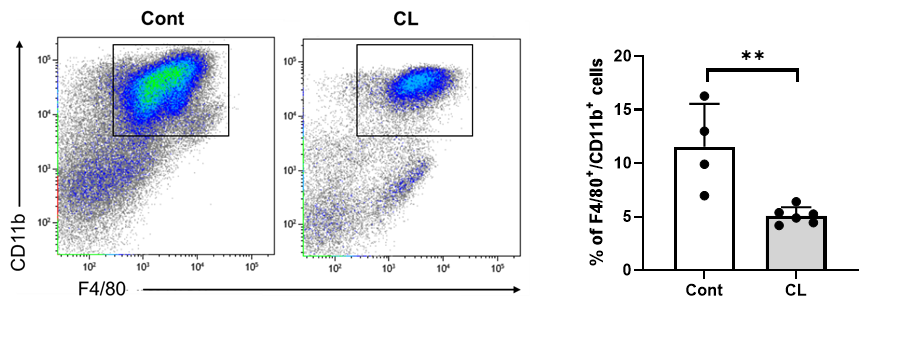


Supplementary Figure 3

Clodronate liposomes (CL) reduced the number of macrophages after APAP treatment.

Representative dot plots of liver macrophages (F4/80^+^/CD11b^+^ cells) gated out CD45^+^/Ly6G^high^/CD11b^high^ cells and the number of macrophages 48 h after APAP treatment in WT mice treated with control liposomes (Cont) and CL. Data are expressed as the mean ± SD (n = 4-6 mice per group). ***p* < 0.01.


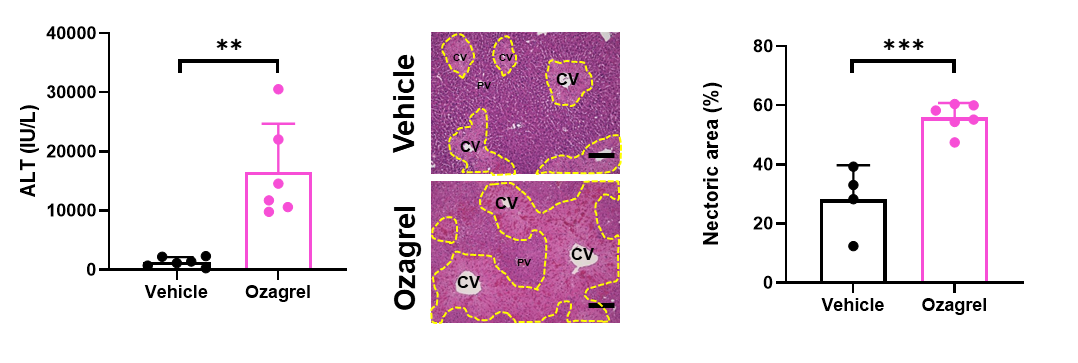


Supplementary Figure 4

The TXS inhibitor Ozagrel aggravated APAP-induced liver injury.

The TXS inhibitor Ozagrel increased the levels of ALT and hepatic necrotic area 48 h after APAP treatment as compared with vehicle. Representative photos of H&E staining of liver sections. CV, central vein. PV, portal vein. Scale bars: 200 μm. Data are expressed as the mean ± SD (n = 4–6 mice per group). ***p* < 0.01, ****p* < 0.001.


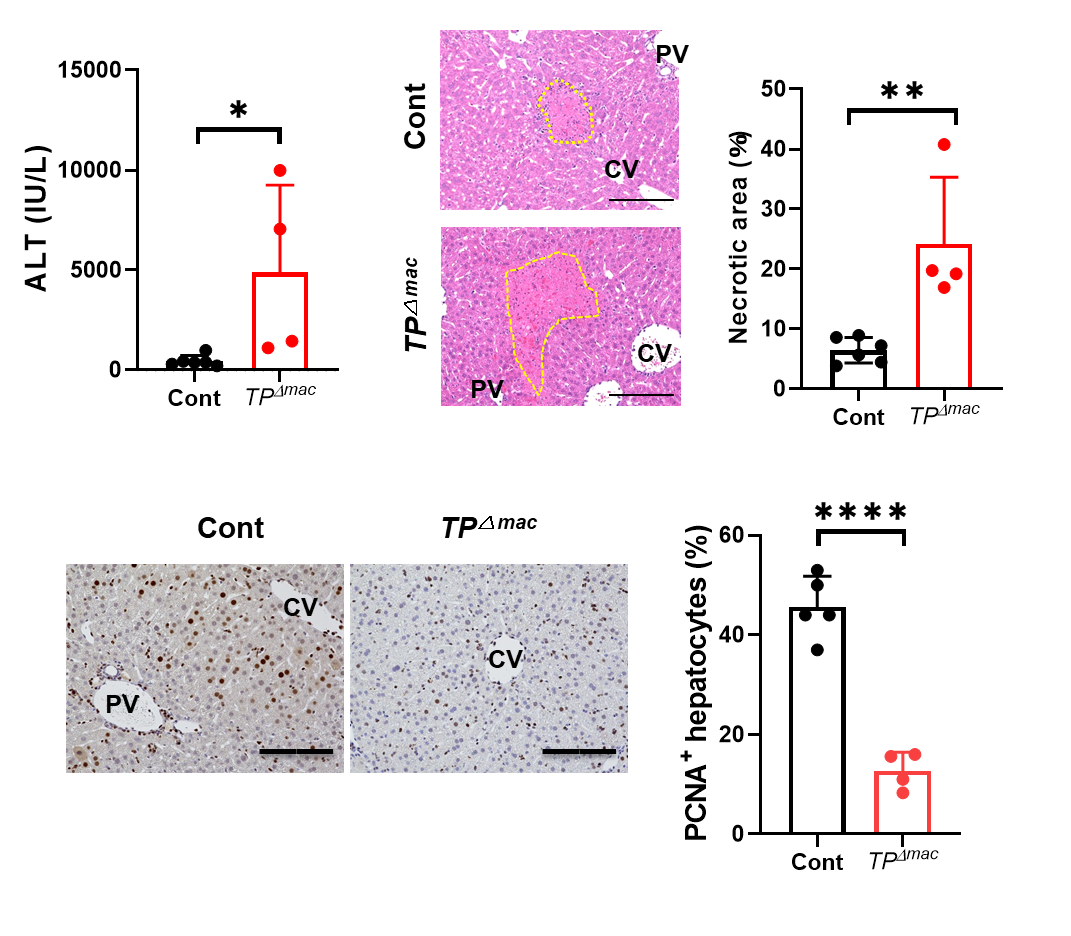


Supplementary Figure 5

Delayed liver repair after concanavalin A (ConA)-induced liver injury in *TP*^△mac^ mice.

The levels of ALT and hepatic necrotic area were increased and PCNA expression was decreased 48 h after concanavalin A (ConA) treatment in *TP*^△mac^ mice as compared with Controls. Representative images of H&E and PCNA staining of liver sections. CV, central vein. PV, portal vein. Scale bars: 200 μm. Data are expressed as the mean ± SD (n = 4–6 mice per group). * *p* < 0.05, ***p* < 0.01, *****p* < 0.0001.


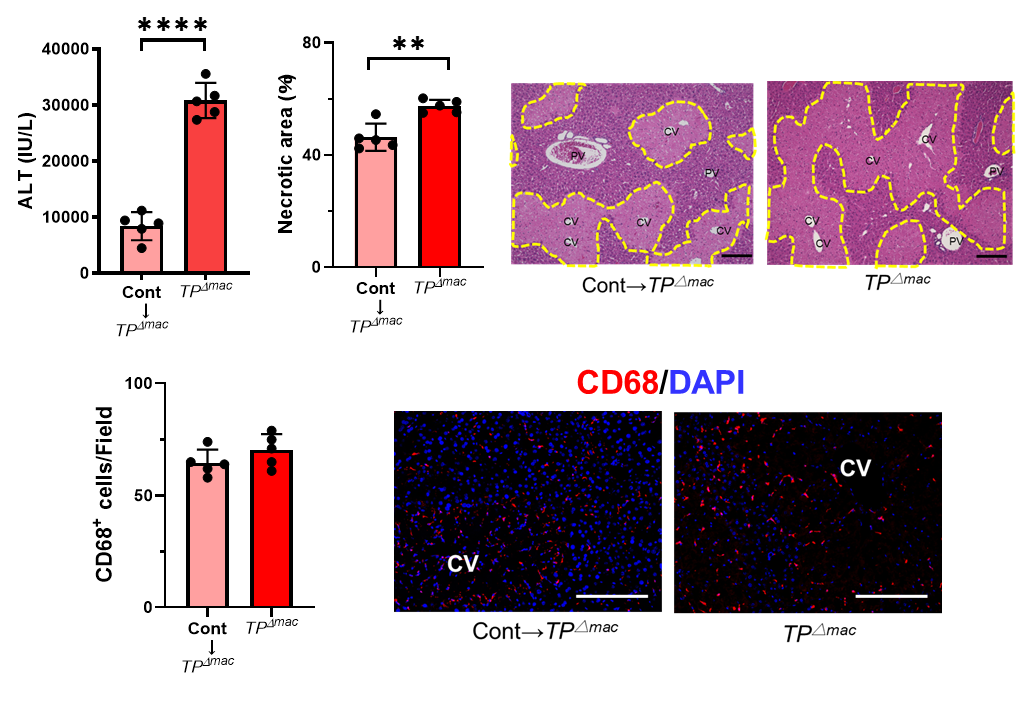


Supplementary Figure 6.

Effects of the adoptive transfer of BM-derived macrophages on APAP-induced liver injury.

ALT levels, hepatic necrotic area, and CD68+ cells 24 h after APAP treatment in *TP*^△mac^ mice with and without the transfer of BM-derived macrophages from Control. BM-derived macrophages were transferred to mice 4 h after APAP administration. Representative photos of H&E and CD68 staining of liver sections. CV, central vein. PV, portal vein. Scale bars: 200 μm. Data are expressed as the mean ± SD (n = 5 mice per group). ***p* < 0.01, *****p* < 0.0001.


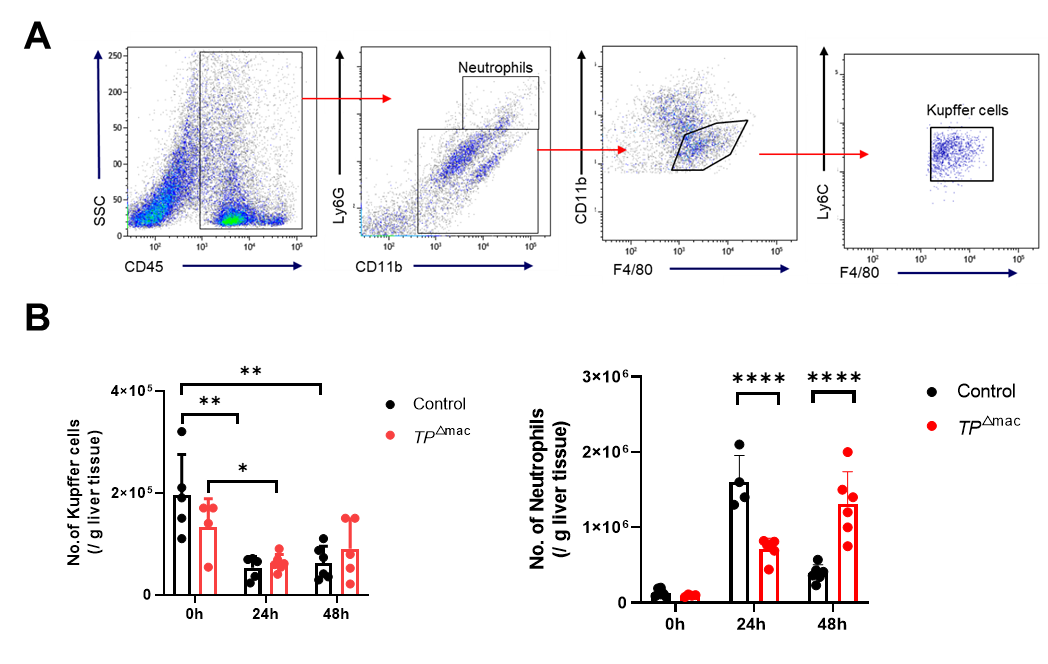


Supplementary Figure 7.

Changes in Kupffer cells and neutrophils in the livers after APAP treatment.

(A) Flow cytometry gating strategy used to identify Kupffer cells and neutrophils in livers from WT mice 0 h after APAP treatment. (B) Changes in the numbers of Kupffer cells and neutrophils after APAP treatment. Neutrophils were defined as Ly6G^+^ and CD11b^+^ cells, and Kupffer cells were defined as Ly6G^-^ / CD11b^int^ /Ly6C^low^/F4/80^high^ -cells. Data are expressed as the mean ± SD (n = 4–6 mice per group). * *p* < 0.05, ***p* < 0.01, *****p* < 0.0001.

Supplementary Table 1: The primers used for reverse transcription and quantitative PCR reactions.

|  |  |  |
| --- | --- | --- |
| *Gene* | Forward primer sequence (5'-3') | Reverse primer sequence (5'-3') |
| *Tp* | CCTCCTGCTCAACACCGTTAG | CTGAACCATCATCTCCACCTC |
| *Txs* | GGATTCTGCCCAATAAGAACC | GAAGTCTCTCCGCCTCTCTTC |
| *Tnfa* | TCTTCTCATTCCTGCTTGTGG | GATCTGAGTGTGAGGGTCTGG |
| *Il1b* | TACATCAGCACCTCACAAGCA | CCAGCCCATACTTTAGGAAGA |
| *Il6* | CAAAGCCAGAGTCCTTCAGAG | TAGGAGAGCATTGGAAATTGG |
| *Mr* | TTTGTCCATTGCACTTTGAGG | TGCCAGGTTAAAGCAGACTTG |
| *Fizz1* | CAAGGAACTTCTTGCCAATCCAG | CCAAGATCCACAGGCAAAGCCA |
| *Ccl2* | CCCAATGAGTAGGCTGGAGAG | GTCTGGACCCATTCCTTCTTG |
| *Ccl7* | TGCTTTCAGCATCCAAGTGTG | ACCGACTACTACTGGTGATCCTTC |
| *Ccl9* | ACAACTGCTCTTGGAATCTGG | AGTCTTGAAAGCCCATTGTGAA |
| *Hgf* | GGCTGAAAAAGATTGGATCAGG | CCAGGAACAATGACACCAAGA |
| *Gapdh* | ACATCAAGAAGGTGGTGAAGC | AAGGTGGAAGAGTGGGAGTTG |
